# A Descriptive Analysis of Morbidity Patterns Among Insured Pet Cats in China Based on Insurance Claims

**DOI:** 10.3390/vetsci13080817

**Published:** 2026-08-17

**Authors:** Kaixin Zhang, Chang Wang, Richard Laven, Baiyu Li, Meng Li, D. Aaron Yang

**Affiliations:** 1 College of Veterinary Medicine, Nanjing Agricultural University, Nanjing 210095, China; zhangkaixin@stu.njau.edu.cn (K.Z.); r.laven@massey.ac.nz (R.L.); 2 Ant Group, Hangzhou 310023, China; xingzhe.wc@antgroup.com (C.W.); baiyu.li@antgroup.com (B.L.); 3 Täwharau Ora–School of Veterinary Science, Massey University, Palmerston North 4442, New Zealand

**Keywords:** feline, epidemiology, morbidity, incidence rate, pet insurance, China

## Abstract

Population-level information on feline morbidity in China remains limited despite the rapid growth of the pet cat population. This study described morbidity patterns among insured cats in China using insurance claims from a major pet insurance provider, with diagnoses derived from veterinary records. Disease events were classified into hierarchical diagnostic categories and subcategories using disease names and clinical descriptions. The study population included 163,890 insured cats contributing 194,284 cat-years at risk. A total of 42,867 disease events were identified in 31,870 cats, and ~ 19.5% of insured cats had at least one disease event during follow-up. The overall incidence rate was ~22 events per 100 cat-years. Digestive disorders were the most commonly claimed disorders, followed by urinary disorders, infectious diseases, respiratory disorders, and dermatological disorders. Gastroenteritis and lower urinary tract disease were the most frequent diagnostic subcategories. Cats aged less than one year had the highest overall incidence rate. Male cats had a higher incidence rate than female cats, mainly reflecting the higher burden of lower urinary tract disorders. Descriptive differences were also observed among British Shorthair, Maine Coon, and Devon Rex cats. This study provides the first broad insurance-based description of feline morbidity across multiple diagnostic categories in China. The findings offer baseline evidence for future disease-specific epidemiological studies.

## Simple Summary

Cats are the most common pets in China, but little is known about the health problems that most often lead owners to seek veterinary care. This study used pet insurance claims from more than 160,000 cats to describe which diseases were most common, how often they occurred, and whether disease patterns differed by age, sex, and breed. Approximately one in five cats had at least one disease event during the study period, with digestive, urinary, infectious, respiratory, and dermatological disorders accounting for the greatest morbidity burden. Gastroenteritis and lower urinary tract disease were the leading diagnostic subcategories. Young cats, especially those less than one year of age, had the highest overall disease rate. Male cats had more urinary disorders than female cats, and differences in disease patterns were also observed among British Shorthair, Maine Coon, and Devon Rex cats. The study shows that insurance data can provide useful information about cat health at a population level, even though the records do not contain all clinical details. These findings can help veterinarians, cat owners, insurers, and researchers better understand common cat health problems, improve preventive care, and guide future studies on feline disease in China.

## 1. Introduction

Cats are among the most widely kept companion animals worldwide, and the pet cat population in China has expanded rapidly in recent years. Recent industry data estimate that urban China had approximately 72.9 million pet cats in 2025, exceeding the number of pet dogs and reflecting the growing importance of feline health in companion-animal practice (China Pet Industry White Paper 2026; https://en.cipscom.com/IndustryNews/25434.htm, accessed on 11 August 2026). Despite this large population, population-level information on the diseases affecting Chinese pet cats remains limited, with previous studies focusing on specific regions [1,2], small numbers of veterinary clinics [3], or particular diseases [4,5], resulting in a lack of data at the population level on how often cats require veterinary care, which diagnostic categories account for the greatest morbidity burden, and how these estimates vary by individual cat-level factors, such as breed, sex, and age.

Such data are valuable because knowledge of morbidity patterns helps veterinarians prioritize differential diagnoses, guide owner education, support preventive health planning, and inform breeding decisions. In this context, the term “morbidity patterns” should be understood not simply as a list of common diseases, but also as the distribution of veterinary care events across diagnostic categories, together with incidence rates, proportional morbidity, and differences among subpopulations. Such information is especially useful when estimates are based on a defined population at risk rather than only on animals presented to individual clinics.

Pet insurance data, which are based on clinical diagnoses, provide a useful source for this type of epidemiological research. Unlike many clinic-based studies, insurance databases can include information on both cats with claims and enrolled cats without claims, allowing time-at-risk to be estimated and incidence rates to be calculated. Previous work has emphasized that insurance data can reveal common, costly, or severe conditions at the population level, although interpretation requires attention to diagnostic accuracy (especially if purely owner reported), insurance coverage, and differences between insured and uninsured animals [6]. To our knowledge, broad insurance-based feline morbidity studies have been conducted primarily in Sweden and Japan, with four key reports forming the basis of comparisons with the present study. In Sweden, Egenvall et al. [7] described morbidity in insured cats from 1999 to 2006. They found that trauma, gastrointestinal disease, and lower urinary tract disease were the leading causes of veterinary care events, with morbidity varying markedly by age, sex, and breed. A later Swedish study using data collected from 2011 to 2016 extended this work by comparing domestic crosses and purebred cats, showing higher overall morbidity in purebred cats [8]. In addition, they identified digestive disease, injury, non-specific systemic signs, skin disease, and urinary disorders as major contributors to feline morbidity. In Japan, Inoue et al. [9], using insurance data from 2008 to 2013, calculated annual incidence rates of 18 diagnostic categories of disease and found that insured cats most commonly claimed for digestive, urinary, and dermatological disorders, with clear variation by age, sex, and breed. A related one-year prevalence study using the data from the same insurance company confirmed similar patterns, reporting a high annual prevalence of urinary, digestive, and dermatological conditions and substantial breed-specific differences [10].

Pet insurance data have also been applied to disease-specific questions in cats, such as dystocia [11], pyometra [12], and periodontal disease [13], and also more general questions, such as antimicrobial exposure [14]. Together, these studies show that insurance data can support both broad morbidity estimates and more specific epidemiological analyses. In China, insurance data have recently been used to study feline lower urinary tract disease (FLUTD), but this work focused on a single disease group rather than the wider distribution of feline morbidity across diagnostic categories [15]. Therefore, population-level evidence on the incidence rates and proportional morbidity of common feline disorders in insured cats in China remains limited apart from anecdotal evidence. International findings may not transfer directly to China because cat demographics, management practices, vaccination uptake, veterinary-care-seeking behavior, and breed composition could differ between countries. Therefore, this study aimed to describe morbidity patterns in insured cats in China using insurance claims from September 2023 to February 2025 from a major pet insurance provider. Specifically, the study (1) estimated overall and cause-specific incidence rates, (2) reported proportional morbidity across diagnostic categories and subcategories, and (3) examined the effect of age, sex, breed, and calendar month on these outcomes. By providing the first broad insurance-based estimates of feline morbidity in China, this study offers baseline evidence for veterinary practitioners, owner education, preventive health planning, and future epidemiological research.

## 2. Material and Methods

### 2.1. Data Source and Study Population

This study used insurance records from a major pet insurance provider in China. The data have been described previously in a study of FLUTD in insured cats in China [15], and thus are summarized only briefly here. The records include cats in 32 province-level administrative regions in China. The observation period extended from 24 September 2023, the earliest insurance enrolment date, to 25 February 2025, the last recorded claim date. The study population comprised only British Shorthair, Maine Coon, and Devon Rex cats, as the three breeds were selected by the insurance company as the population of interest for this study. Cats of other breeds were not included and, therefore, outside the scope of this analysis. All cats included in this study had active insurance coverage throughout this entire observation period. The available variables included breed, sex, neuter status, date of birth, insurance enrolment date, claim date, location, disease name, and claim description (clinical notes). Each cat was individually identified using a unique identifier.

### 2.2. Diagnostic Classification

A disease event was defined as one episode of illness identified from one or more related claims (an insurance reimbursement record submitted for veterinary care). The disease events were classified based on both the disease name and the claim description using manual record classification. In general, the classification dictionary, hierarchical categories, and priority rules were jointly developed by K.Z., M.L., and D.A.Y. Individual records were subsequently classified manually by K.Z. The classification system was refined iteratively during record review. Diagnostic terms or clinical descriptions that were not adequately addressed by the initial rules were identified and incorporated into the classification dictionary. The revised rules were then applied consistently to all relevant records. Records related to non-disease conditions, such as elective neutering, were excluded from disease classification. A hierarchical diagnostic classification system was developed to convert the raw claim diagnoses into structured diagnostic categories (level 1) and subcategories (level 2). Word frequency analysis was first performed on the disease names (in Chinese) to identify commonly occurring disease terms, with terms occurring at least 300 times used to guide the initial grouping of diagnoses by organ system (e.g., digestive, urinary, respiratory, and musculoskeletal), anatomical site (e.g., eye, ear, and mouth), and disease process (e.g., infectious disease, trauma, and neoplasia). For records with non-specific diagnostic labels, such as diarrhea, vomiting, anorexia, lethargy, infection, or inflammation, the claim description (in Chinese) was used to identify the affected organ system, anatomical site, or disease process where possible. For example, records labeled as infection or inflammation were classified as digestive system disorders if the claim description indicated intestinal or pyloric involvement, as dermatological disorders if the claim description indicated skin lesions or pruritus, and as urinary disease if the claim description included signs such as frequent urination, dysuria, or hematuria. If neither the diagnosis nor the description provided sufficient information to identify a specific disease process or anatomical site, the record was classified as “non-specific”. Disease names occurring fewer than 300 times were subsequently reviewed and assigned to the existing diagnostic categories using the same classification rules with reference to the claim description when necessary. When a record met the criteria for more than one diagnostic category, predefined priority rules were applied to assign a single final category. Neoplasia and infectious diseases were prioritized over organ system or anatomical site categories. Organ system and anatomical site categories were prioritized over trauma. When more than one anatomical site category was applicable, the more specific site was prioritized, for example, records referring to ear mites were classified as ear disease rather than dermatological disorders.

After assignment to a diagnostic category, records were further grouped into diagnostic subcategories according to the specific diagnosis, or similar clinical conditions. For example, urinary disorders were divided into lower/upper urinary tract, and respiratory disorders included lower/upper respiratory (non-specific), pneumonia, tracheitis, and “other” respiratory disorders. The claim description was also used when the disease name was non-specific in order to determine the subcategory, for example, records labeled as infection were assigned to subcategories, such as feline calicivirus infection (FCV), feline infectious peritonitis (FIP), and feline herpesvirus type 1 infection (FHV-1), when the description provided corresponding laboratory-confirmed diagnostic information.

### 2.3. Covariates and Time-at-Risk

Breed was recorded as British Shorthair, Maine Coon, or Devon Rex. Sex was coded as male or female. Neuter status (intact vs. neutered) was based on the status recorded at insurance enrolment, because updated neuter status during follow-up was not available. For each cat, time-at-risk began on the insurance enrolment date. The end of time-at-risk was defined as the recorded death date, if available, or, if it was not available, 25 February 2025. Cats without disease claims, including cats with no claims and cats with claims related only to non-disease conditions, remained in the population-at-risk and contributed cat-time to the denominator, but did not contribute disease events to the numerator. For each cat, claim records were sorted by claim date. Repeated claims for the same disease (identical recorded diagnosis) within two weeks of the previous claim were treated as follow-up records for the same disease episode and were not counted as new disease events. The earliest claim in each such episode was retained as the event date.

**Figure 1:**
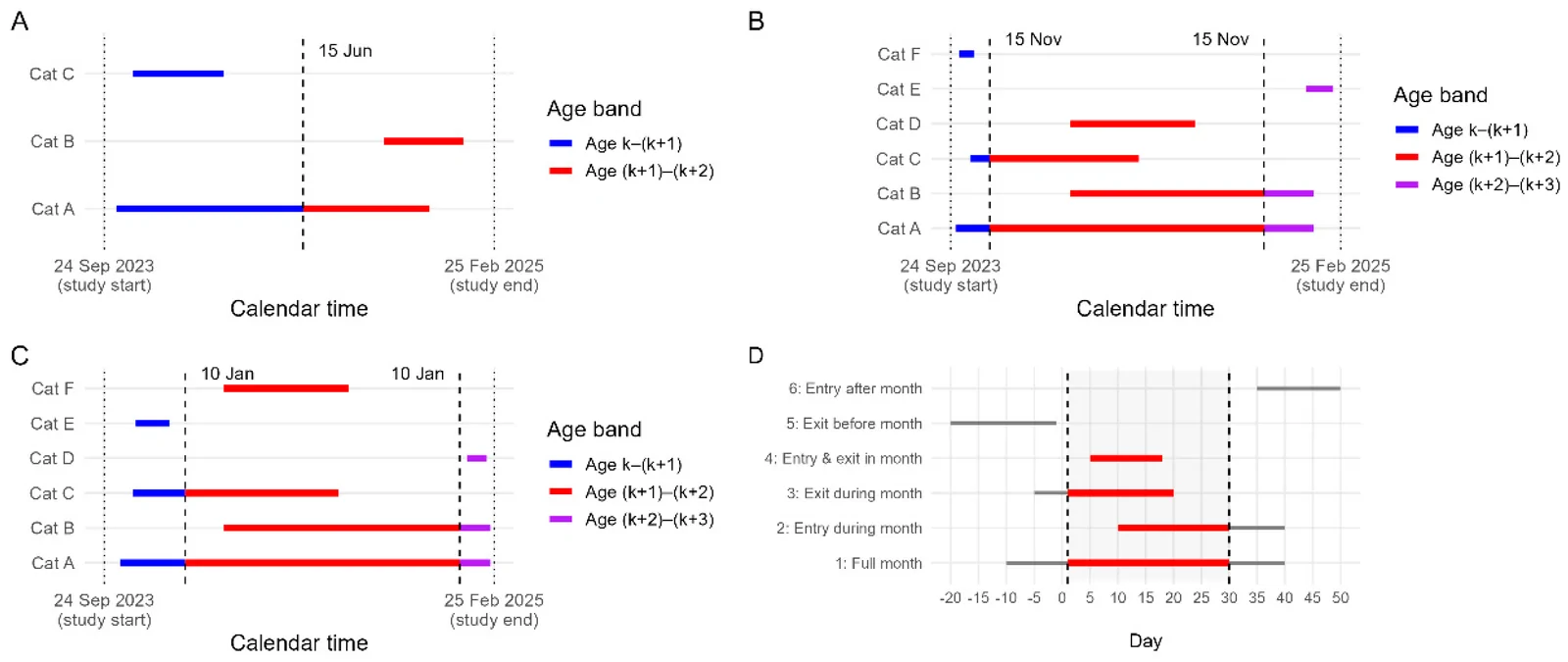
Hypothetical follow-up patterns used to calculate time-at-risk across age groups and calendar months for insured cats during the study window from 24 September 2023 to 25 February 2025. (**A**) A cat aged k at insurance enrolment with a birthday on 15 June, representing cats born between 26 February and 23 September, has one birthday within the study window and contributes time-at-risk to both the age k and age k + 1 groups. (**B**) A cat aged k at enrolment with a birthday on 15 November, representing cats born between 24 September and 31 December, may have birthdays in both 2023 and 2024 and, therefore, contributes time-at-risk to the age k, age k + 1, and age k + 2 groups. (**C**) A cat aged k at enrolment with a birthday on 15 January, representing cats born between 1 January and 25 February, may have birthdays in both 2024 and 2025 and contributes time-at-risk to the age k, age k + 1, and age k + 2 groups. (**D**) Six possible patterns of overlap between an individual cat’s observation period and a calendar month, illustrating how time-at-risk was allocated to the corresponding month.

Age was treated as a time-varying covariate based on attained age. Age groups were defined in completed years of age, for example, the age0 group included cat-time from birth to <1 year, the age1 group included cat-time from 1 to <2 years, and so forth. For each cat, time-at-risk was split at dates of birth, and each segment of cat-time was allocated to the corresponding age group. Thus, for example, for a cat that became 3 years old during the observation period, the time before the third birthday contributed to the age2 group, whereas time after the third birthday (including the birthday) contributed to the age3 group. Disease events were assigned to the age group according to the claim date and birthday. The number of birthday cut points in the observation period (24 September 2023 to 25 February 2025) depended on the cat’s birth date. Three scenarios were, therefore, used to illustrate the allocation of cat-time: (1) cats with birth dates between 26 February and 23 September could have at most one birthday within the observation period, (2) cats with birth dates between 24 September and 31 December could have birthdays in both 2023 and 2024, and (3) cats with birth dates between 1 January and 25 February could have birthdays in both 2024 and 2025. Within each scenario, the hypothetical follow-up patterns are enumerated to illustrate how a cat’s insurance enrolment and end-of-time-at-risk dates determined the allocation of time-at-risk across age groups (Figure 1A–C).

Month was also treated as a time-varying variable. Each cat’s follow-up time was divided at the start and end of each calendar month, so that the time under observation within a given calendar month contributed to the time-at-risk for that month. Hypothetical follow-up patterns are enumerated to illustrate how cats enrolled before, during, or after a hypothetical month (with 30 days) contributed full, partial, or no time-at-risk to that month (Figure 1D). Disease events were assigned to the month when the claim occurred.

### 2.4. Statistical Analysis

Data processing and statistical analyses were conducted in R version 4.5.0. Incidence rates were calculated as the total number of disease events divided by cat-years at risk and expressed per 10,000 cat-years. Overall incidence rates as well as the stratified incidence rates for diagnostic categories and subcategories were calculated by breed, sex, neuter status, age groups, month, and a combination of age and sex, with the Wald 95% confidence interval (95%CI) computed by assuming a Poisson distribution of event counts [16]. For age-specific and month-specific incidence rates, denominators were based on the split cat-time described above. Numerators were based on disease events assigned to the corresponding age group or month. Proportional morbidity for each diagnostic category was calculated as the number of cats with at least one disease event in that category divided by the total number of cats with at least one disease event. Because individual cats could have claims in multiple diagnostic categories or subcategories, proportional morbidity estimates were not mutually exclusive.

## 3. Results

### 3.1. Study Population and Diagnostic Classification

The study population comprised 177,517 records generated from 163,890 insured cats contributing 194,284 cat-years at risk between 24 September 2023 and 25 February 2025. The median follow-up was 427 days. A total of 114 cats were censored due to death, accounting for 0.07% of all censored observations, while the remaining 163,776 cats were censored at the end of the study. During the observation period, 42,867 (new) disease events were identified in 31,870 cats. Among cats with disease events, 24,032 (75.4%) had 1 recorded disease event, 5698 (17.9%) had 2, 1503 (4.7%) had 3, and 637 (2%) had 4 or more. Disease events were classified into diagnostic categories and subcategories using the hierarchical classification system. The 17 diagnostic categories were digestive disorders, urinary disorders, infectious diseases, respiratory disorders, dermatological disorders, trauma, non-specific clinical signs, ocular disorders, otic disorders, oral disorders, reproductive disorders, immunological disorders, cardiovascular and hematological disorders, musculoskeletal disorders, neoplasia, neurological disorders, and metabolic and endocrine disorders.

### 3.2. Disease Burden by Breed, Sex, and Neuter Status

Overall, 19.5% (95%CI: 19.3–19.6%) of insured cats had at least one disease event during the follow-up. The overall incidence rate was 2206 events per 10,000 cat-years (95%CI: 2185–2227). The distribution of breed, sex, and neuter status is shown in Table 1. British Shorthair cats represented the largest proportion of the study population, accounting for 89.4% of insured cats, but had the lowest incidence rate among the three breeds: 2088 events per 10,000 cat-years (95% CI: 2066–2109). Male cats accounted for 62.3% of the population and had a higher incidence rate of any disease than female cats 2414 (95%CI: 2386–2441) vs. 1865 (95%CI: 1833–1896) events per 10,000 cat-years, respectively). At insurance enrolment, 41.5% of cats were recorded as neutered, and the incidence rate was 2113 events per 10,000 cat-years in neutered cats (95%CI: 2081–2145) and was slightly lower than that of in intact cats (2272 events per 10,000 cat-years; 95%CI: 2244–2300).

### 3.3. Age- and Month-Specific Incidence Rates

**Figure 2:**
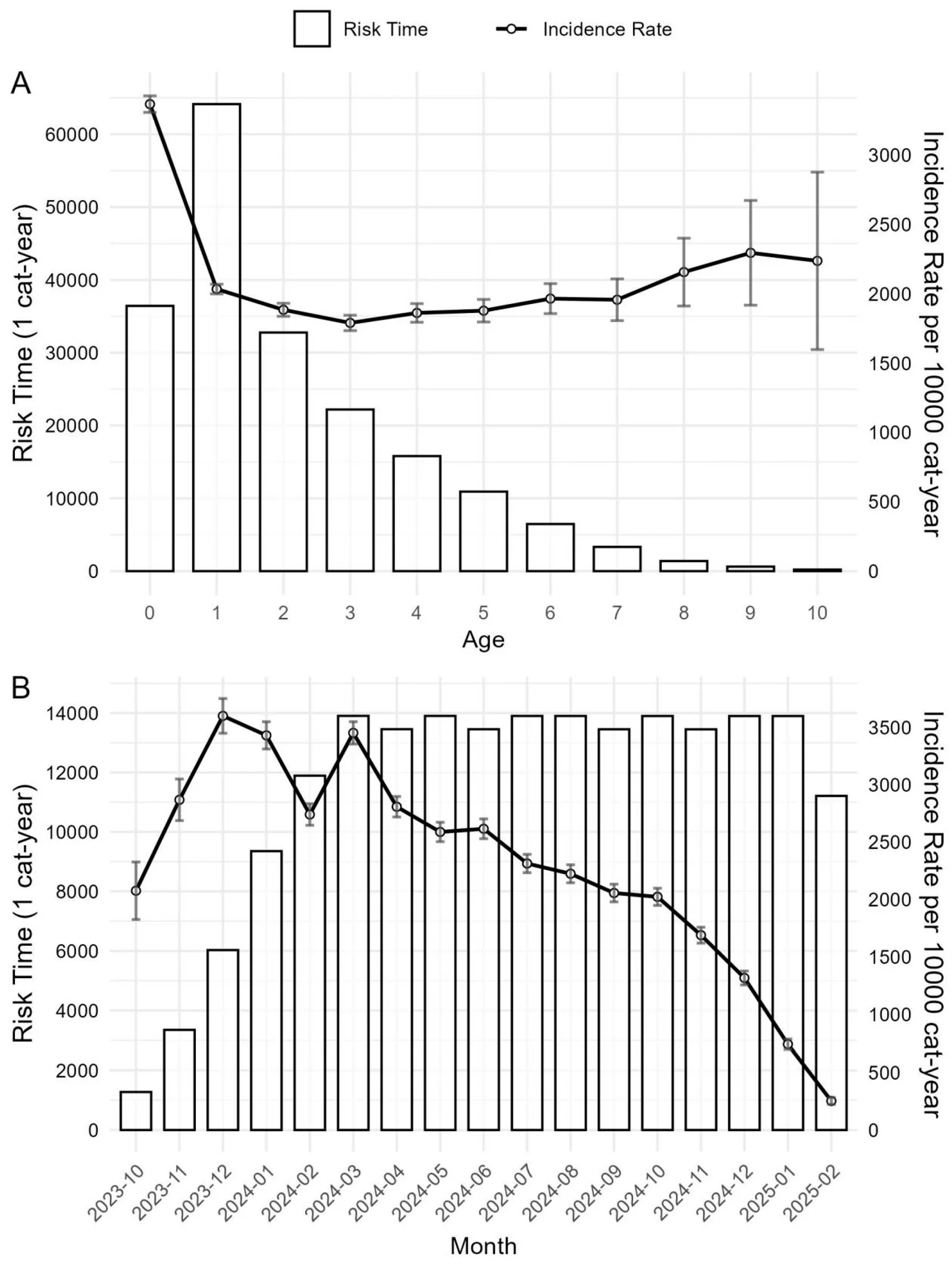
Incidence rates and time-at-risk by age group and calendar month among insured cats with claims recorded between September 2023 and February 2025. Incidence rates are expressed per 10,000 cat-years, and bars indicate time-at-risk in cat-years. (**A**) Age-specific incidence rates and time-at-risk. Age groups were defined in completed years: age 0 included cat-time from birth to <1 year, age 1 included cat-time from 1 to <2 years, and so forth. (**B**) Monthly incidence rates and time-at-risk.

Age-specific incidence rates are shown in Figure 2A. Cats aged less than 1 year had the highest incidence rate of any disease, at 3368 events per 10,000 cat-years (95%CI: 3308–3427). This incidence rate decreased in cats aged 1 year, to 2034 events per 10,000 cat-years (95%CI: 1999–2069), and remained relatively stable from ages 1 to 10 years. Monthly incidence rates varied during the observation period (Figure 2B). The highest monthly rates occurred between December 2023 and March 2024, ranging from 2740 (95%CI: 2646–2834) to 3600 (95%CI: 3448–3751) events per 10,000 cat-years. The incidence rate decreased continuously from March 2024 to the lowest level in February 2025 at 252 events per 10,000 cat-years (95%CI: 223–282).

### 3.4. Diagnostic Category- and Subcategory-Specific Incidence Rates and Proportional Morbidity

The incidence rates and proportional morbidity for the 17 diagnostic categories are shown in Table 2. Digestive disorders had the highest incidence rate, at 644 events per 10,000 cat-years (95%CI: 632–655), followed by urinary disorders at 458 per 10,000 cat-years (95%CI: 449–468), infectious diseases at 222 per 10,000 cat-years (95%CI: 216–229), respiratory disorders at 208 per 10,000 cat-years (95%CI: 201–214), and dermatological disorders at 201 per 10,000 cat-years (95%CI: 195–208). These categories also accounted for the largest proportions of affected cats (a unique cat with at least one disease event during the study period). Among cats with at least 1 disease event, proportional morbidity, calculated using the 31,870 unique affected cats as the denominator, was 34.1% for digestive disorders, 23.8% for urinary disorders, and more than 10% each for infectious diseases, respiratory disorders, and dermatological disorders.

Diagnostic subcategory results are summarized in Table 3. Gastroenteritis was the main contributor to digestive disorders, accounting for 60.9% of digestive disease events. Lower urinary tract disease accounted for 95.0% of urinary disease events and was the most frequent diagnostic subcategory overall. Feline calicivirus infection and feline infectious peritonitis were the most frequent infectious disease subcategories, while lower respiratory disorders were the most frequent respiratory subcategories. Overall, lower urinary tract disease and gastroenteritis were the two most common diagnostic subcategories.

### 3.5. Age*Sex and Breed-Specific Diagnostic Patterns

**Figure 3:**
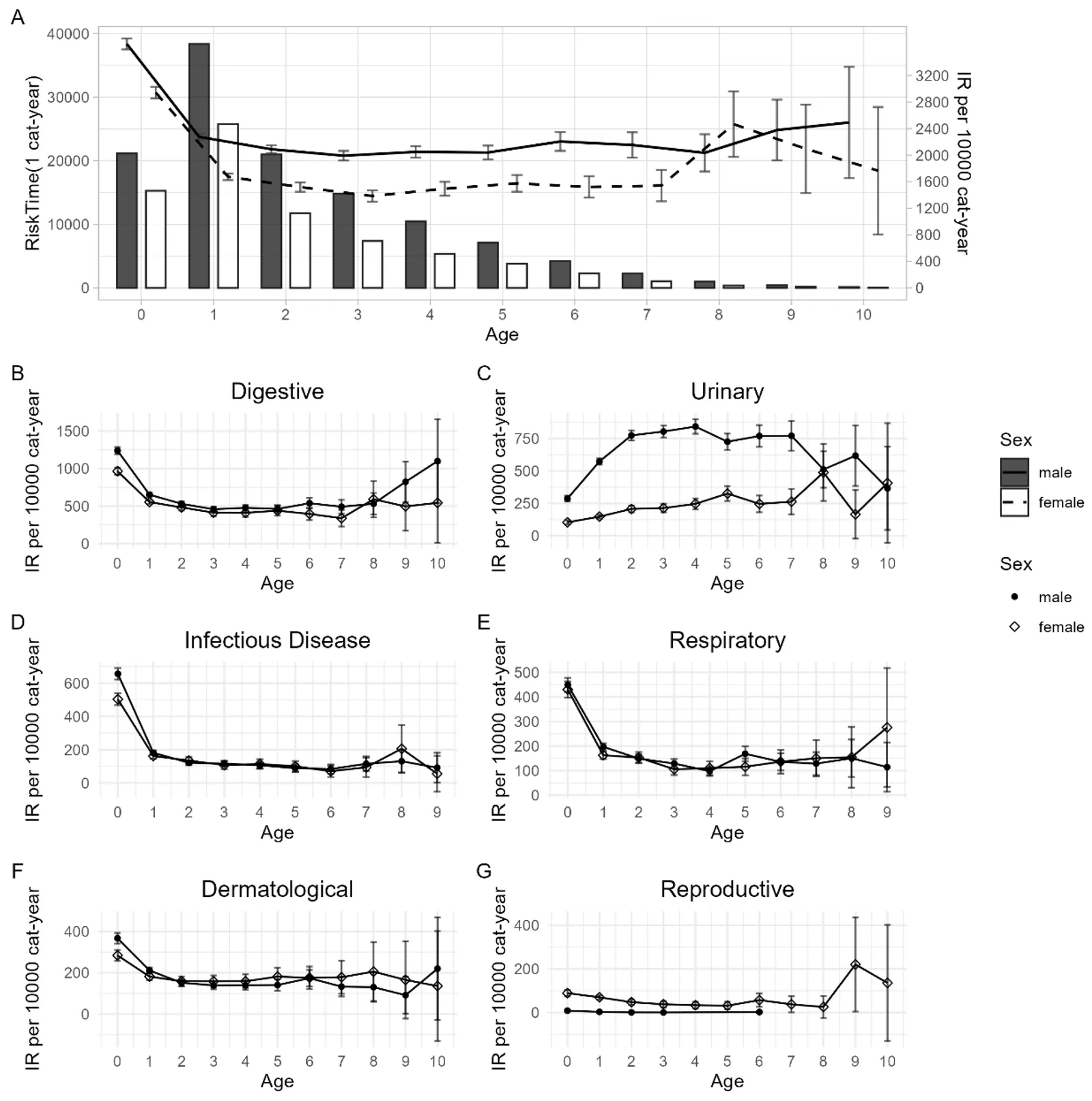
Age- and sex-specific incidence rates with 95% confidence intervals among insured cats. Incidence rates are expressed per 10,000 cat-years. Age groups were defined in completed years: age 0 included cat-time from birth to <1 year, age 1 included cat-time from 1 to <2 years, and so forth. (**A**) Overall age- and sex-specific incidence rates. (**B**–**G**) Age- and sex-specific incidence rates for digestive, urinary, infectious disease, respiratory, dermatological, and reproductive diagnostic categories.

**Figure 4:**
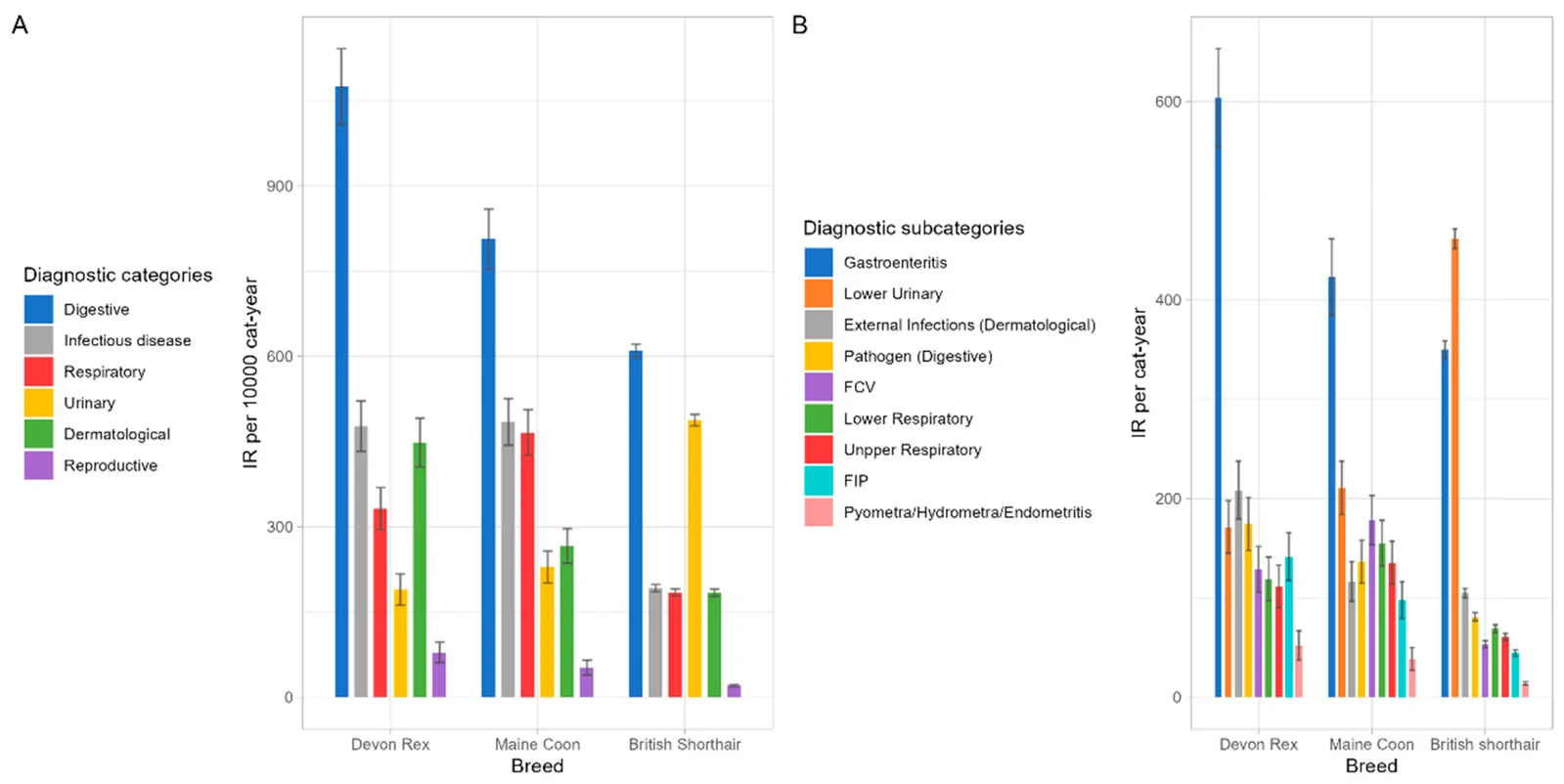
Incidence rates across three breeds among insured cats. Incidence rates are expressed per 10,000 cat-years with 95% confidence intervals. (**A**) Breed-specific incidence rates for three breeds across six selected diagnostic categories. (**B**) Breed-specific incidence rates for three breeds across nine selected diagnostic subcategories. The category “lower respiratory” excludes pneumonia.

Age*sex patterns are shown in Figure 3. Sex differences were most apparent for urinary and reproductive disorders. Reproductive disorder incidence was higher in female cats, whereas urinary disorder incidence was higher in male cats. Among the five most common diagnostic categories (i.e., digestive disorders, urinary disorders, infectious diseases, respiratory disorders, and dermatological disorders), incidence rates were generally highest in age group 0 cats, with the exception of urinary disorders. Age-specific incidence rates across the five most common diagnostic categories and reproductive disorders are shown in Figure 3B–G. Breed-specific incidence rates for the five most common diagnostic categories and selected diagnostic subcategories are shown in Figure 4A,B. Digestive disorders, particularly gastroenteritis, occurred frequently across all three breeds (digestive incidence rate in Devon Rex: 1075 events per 10,000 cat-years, 95%CI: 1009–1041; Maine Coon: 806 events per 10,000 cat-years, 95%CI: 754–859; British Shorthair: 610 events per 10,000 cat-years, 95%CI: 598–622). Devon Rex (477 events per 10,000 cat-years; 95%CI: 433–521) and Maine Coon (484 events per 10,000 cat-years; 95%CI: 444–525) cats had relatively high rates of infectious diseases compared with British Shorthair (192 events per 10,000 cat-years; 95%CI: 185–198). Among the three breeds, Devon Rex cats also had the highest rate of dermatological disorders (448 events per 10,000 cat-years, 95%CI: 405–491), whereas Maine Coon cats had the highest rate of respiratory disorders (466 events per 10,000 cat-years, 95%CI: 426–506). British Shorthair cats had the highest rate of urinary disorders (488 events per 10,000 cat-years, 95%CI: 477–498), especially lower urinary tract disease (462 events per 10,000 cat-years, 95%CI: 452–472), but the lowest incidence rates for most other diagnostic categories and subcategories. Breed-specific incidence rates for all diagnostic categories are available in the Supplementary Materials.

## 4. Discussion

To our knowledge, this is the first broad insurance-based study to describe feline morbidity across multiple diagnostic categories in China using claims derived from veterinary insurance records. The key advantage of this type of data is that we have a defined population that allows cats with and without disease claims to contribute time-at-risk, making it possible to estimate incidence rates rather than simply the distribution of diagnoses, which would be the case had we used data on cats presented to veterinary clinics. However, this comes with the limitation that the diagnoses are not as detailed as would be the case if we prospectively collected data at veterinary clinics. Nevertheless, the results provide baseline information on the disorders most frequently recorded in insured cats in China and identify several patterns that warrant further investigation.

In our dataset, digestive and urinary disorders accounted for the largest morbidity burden (34.1% and 23.8% of all disorders, respectively). These are well-recognized components of feline morbidity internationally [17,18], but both the relative and absolute burdens in this dataset are different from previous reports based on insurance data (Table 4). Since our primary incidence rates were calculated using the total number of disease events, whereas previous studies counted cats with at least one event in a diagnostic category, we re-calculated a modified incidence rate for cross-study comparison according to the definition used in the previous studies [7,8,9,10]. For simplicity, subsequent cross-study comparisons are based on the modified incidence rate, without further distinguishing it from the original event-based incidence rate. Using this comparable measure, digestive and urinary disorders were higher in the current Chinese dataset than in the Swedish studies. Proportional morbidity was also higher in the current dataset than in Sweden for both categories (see Table 4). In contrast, the Japanese studies showed lower proportional morbidity for digestive disorders and similar or slightly lower proportional morbidity for urinary disorders, but substantially higher modified rates. Inoue et al. (2016) reported rates of 1172 and 1091 per 10,000 cat-years for digestive and urinary disorders, respectively, while the one-year prevalence estimates from Isomura et al. (2017), expressed on a comparable per 10,000 cat-year scale, were 1120 for both categories (see Table 4). These differences highlight the limitations of focusing on proportional morbidity when looking at disease data, as similar proportional morbidity can hide markedly different incidences of disease. In this case, the reason for the limited association between proportional morbidity and incidence rate appears to be driven partly by large differences in the overall burden of claimed disease. In the Japanese datasets, the all-disorders rate is over 4600 disease events per 10,000 cat-years, while in the Swedish datasets the equivalent rate ranges from 875 to 1430 disease events per 10,000 cat-years. In the current Chinese dataset, the overall rate is above the Swedish rates but still considerably lower than the Japanese rates. These large differences are almost certainly related, at least in part, to differences in the population demography—both the Japanese and Chinese populations have much higher proportions of pedigree cats than is present in the Swedish population and they are much more likely to be kept permanently indoors, 93.9% in Japan [9] and 73.5% in urban China [19], but it may also reflect differences in the likelihood of a cat being taken to a veterinarian, particularly for relatively minor complaints. Assessing the importance of such differences in claim behavior on incidence rates requires more detailed information than is present on insurance forms. Good quality veterinary practice records could provide such information, but without linking these records to insurance data we lose the clear denominator needed to calculate the incidence rates that we are trying to explain.

The pattern seen in the data for digestive and urinary disorders, i.e., Japan much higher than Sweden with China between the two, was also seen for eye, ear, and respiratory disorders. For eye problems, the rate in the current Chinese dataset was higher than that reported in Sweden, but far lower than that reported in Japan. For example, the incidence rate at comparable scale was 72 in China vs. 40–50 per 10,000 cat-years in Sweden [6,8]. Ear disorders showed a larger contrast—the Chinese rate was at least twice as high as the Swedish estimates, but less than 1/10 of the Japanese estimates [6,8,9]. For respiratory disease, the Chinese rate was also approximately four to five times higher than the Swedish estimates but remained approximately half of that reported in Japan [6,8,9]. As with gastrointestinal and urinary disorders these differences across countries are likely to be related at least in part to differences in the insured population and the management of that population. However, to fully understand how these factors interact to affect the risk of disease requires a better understanding of the risk factors for these diseases. For example, a recent scoping review of feline respiratory disease (the most common and important of these disorders) concluded that the lack of a uniform definition and diagnosis of clinical respiratory disease, combined with inconsistent inclusions and definitions of risk factors, means that we lack good quality risk factor information even in high-risk cat populations (such as cat shelters) [20]. Trauma/injury was less common in this dataset than in the Swedish studies, with the incidence rate being ~100 per 10,000 cat-years vs. 174 and 206 for Egenvall, Nødtvedt, Penell, Gunnarsson, and Bonnett [6] and Hadar, Bonnett, Poljak, and Bernardo [8], respectively. It might be, though, that this reduction was down to increased housing of Chinese cats, but in the Japanese populations (which also have high rates of permanently housed cats) the incidence rate of trauma was >300 per 10,000 cat-years. It is unclear what is driving this large difference between China and Japan. In our Chinese dataset, we do have subcategories for trauma (see Table 3), with accident (falling: 652 events, 94.2% of fall/vehicle trauma; road traffic: 40 events, 5.8% of fall/vehicle trauma) accounting for approximately 1/3 of all trauma events, but neither of the published Japanese reports provide such detailed information [9,10].

Of all the diseases in our Chinese dataset that had an incidence rate > 100 per 10,000 cat-years, only infectious disease had an incidence rate that was largest in Chinese cats. Incidence rates were lowest in Swedish cats (33 and 27 for Egenvall, Nødtvedt, Penell, Gunnarsson, and Bonnett [6] and Hadar, Bonnett, Poljak, and Bernardo [8], respectively), but even though Japanese incidence rates were much higher than those in Sweden (170 and 192 for Isomura, Yamazaki, Inoue, Kwan, Matsuda, and Sugiura [10] and Inoue, Hasegawa, and Sugiura [9], respectively), they were still not as high as in our Chinese population (204). A recent veterinary hospital-based study in Shenzhen, China, also identified a substantial burden of common feline infections and reported higher risks among unvaccinated cats [21]. Three of the most frequently recorded infection-causing pathogens in the present study, FCV, FHV-1, and FPV, are targeted by routine core feline vaccines. Recent data from China estimated that the proportion of routinely immunized cats was 48.6% (https://hslcs.org.cn/index.php/info/19645.html, accessed on 11 August 2026). In contrast, vaccination against FCV, FHV-1, and FPV in Sweden is very common with “outbreaks of infectious disease … generally limited to … stray cat populations” [22], and vaccination against FPV is essential to get veterinary insurance [7]. Thus, although the Chinese vaccination data are not necessarily representative of the cats included in our dataset, the comparatively low vaccination uptake reported in China may, at least in part, be responsible for the higher incidence of vaccine-preventable infections compared to Sweden. However, feline vaccination rates in Japan are reportedly very low, with some reports suggesting it may be as low as 10% (https://wsava.org/wp-content/uploads/2020/01/Vaccination-Guidelines-Article-2-Michael-Day-Japanese.pdf, accessed on 11 August 2026). The available insurance data do not tell us whether individual cats were vaccinated, so we cannot directly assess the role of vaccination in infectious disease risk. If broader reported vaccination rates are used only as a rough qualitative indicator, the higher infectious disease rate in the current Chinese dataset suggests that vaccination is unlikely to be the only explanation. Other factors, including household cat density, breed composition, exposure patterns, and veterinary-care-seeking behavior, may also influence infectious disease incidence. This reinforces the need to collect additional background information alongside insurance claims to better interpret morbidity patterns.

Other key differences between these insurance datasets involve diseases that are commonly recorded in primary care studies from other countries but less so in our Chinese insurance-based datasets. For example, ectoparasite infestations accounted for only 1.9% of dermatological disorders (65 events from 65 cats) reported, whereas flea infestation is commonly reported in primary care studies from Europe and North America [23,24,25,26]. This difference may, again, partly reflect variation in outdoor exposure, as outdoor access is common among pet cats in the UK and the USA [27,28], whereas, as discussed earlier, pet cats in urban China are predominantly housed indoors [19].

Another disease commonly reported in primary care studies is obesity [25,29,30]. This was not recorded in the present claims data. This is likely to reflect claim eligibility (i.e., owners will claim for diseases that are the consequence of obesity rather than obesity itself) rather than the absence of obesity among cats in China. This again emphasizes the limitations of insurance-claim-based data when one is trying to identify issues beyond the principal disease category/subcategory responsible for the claim.

The final disease category where our insurance-based dataset does not seem to reflect primary care studies is dental disease. Dental and related problems are very common disorders in cats presented to primary care clinics. For example, O’Neill, Gunn-Moore, Sorrell, McAuslan, Church, Pegram, and Brodbelt [25] reported that >23% of clinical cases presented to a group of veterinary practices involved either periodontal or dental disease in one year. In contrast, in our Chinese dataset the combined incidence rate for these two disorders is only 16/10,000 cat-years (accounting for <1% of all disease events), so they are less common than musculoskeletal disease, immunological disease, and pancreatitis (see Table 2 and Table 3). It is not only our insurance-based dataset in which dental disease is much less frequently recorded than expected. In Sweden, Egenvall, Bonnett, Häggström, Ström Holst, Möller, and Nødtvedt [7] reported that dental disease had an incidence rate of 13 (2% proportional morbidity), while Hadar, Bonnett, Poljak, and Bernardo [8] did not include oral or dental disease in their list of diagnostic categories. Both of the Japanese studies reported much higher incidence rates (> 200) of dental disorders than either the current study or that of Egenvall, Bonnett, Häggström, Ström Holst, Möller, and Nødtvedt [7]; nevertheless, the proportional morbidity (~5% in both Isomura, Yamazaki, Inoue, Kwan, Matsuda, and Sugiura [10] and Inoue, Hasegawa, and Sugiura [9]) was much lower than the 23% reported by O’Neill, Gunn-Moore, Sorrell, McAuslan, Church, Pegram, and Brodbelt [25]. It is likely that this reflects the different populations, with insured cats being younger than the population of cats presented to primary care clinics and dental disease being much more common in older cats [25]. The median age of the cats presented by O’Neill, Gunn-Moore, Sorrell, McAuslan, Church, Pegram, and Brodbelt [25] was 5.7 years, whereas our median age at enrolment was 1.95 years, and that for cats with claims was 1.86 years.

The relative lack of old cats in our dataset is reflected in our analysis of the impact of age on disease risk. Across our insured population, cats less than one year old had the highest overall incidence rate of disorders. The increased disease burden in kittens was evident across several common diagnostic categories, including digestive, infectious, dermatological, and respiratory disorders. This may reflect greater biological susceptibility in kittens, including the transition from maternally derived immunity to active immune development, as well as the high frequency of respiratory and gastrointestinal conditions during early life [9,31]. Incidence rates remained relatively stable after the first year of life and appeared to increase slightly in some older age groups. However, as our population of older cats was relatively small, estimates for older cats should be interpreted cautiously because fewer cats contributed time-at-risk, resulting in wider confidence intervals. Our data also found differences in monthly incidence rates during the observation period with a pronounced decline over the last eight months (Figure 2B). It is unclear what was driving this change. It does not appear to be seasonal in nature, because the monthly incidence rates from October 2024 to February 2025 were all consistently lower than the incidence rates calculated for the months from October 2023 to February 2024. Further data from this population are required to establish whether this pattern is a permanent change or just a transient decrease.

Male cats had a higher overall incidence rate than female cats. This difference appeared to be driven largely by urinary disorders. Lower urinary tract disease accounted for 95% of urinary disease events and occurred more frequently in male cats, consistent with findings from almost all previous studies [15,32]. In contrast, reproductive disorders were more frequently recorded in female cats, predominantly because of pyometra, hydrometra, and endometritis. These findings highlight the value of sex-specific estimates for informing targeted owner education and preventive care.

Differences were observed among the three recorded breeds. British Shorthair cats accounted for most of the insured population but had the lowest overall incidence rate among the three breeds, despite having the highest incidence rate of urinary disorders, particularly lower urinary tract disease. Devon Rex cats had highest incidence rate of digestive and dermatological disorders, whereas Maine Coon cats had the highest rates of respiratory disorders. Previous studies have also reported variation in morbidity among breeds, but the direction and magnitude of these differences have not been consistent across countries [8,9,24]. This inconsistency highlights the need for breed-focused epidemiological studies in China, if we are to provide breed-specific preventive care, owner education, and support breeding decisions that are targeted at the Chinese cat population. However, the present study was restricted to three selected breeds, and further studies including a broader range of breeds are needed to characterize morbidity patterns across the wider cat population in China.

As identified throughout this discussion, the major limitation in this study is that insurance claims are not collected primarily for research purposes [33,34]. Diagnoses are derived from often limited veterinary records submitted for insurance claims rather than from standardized examinations or uniform diagnostic protocols. Thus, although disease names and clinical descriptions were used together to improve classification, the level of diagnostic detail varied greatly among records. Some claims contained specific diagnoses, whereas others used broader clinical labels, such as infection, inflammation, vomiting, or respiratory tract infection. This is likely to have resulted in our classification being more subjective than we would have liked, particularly for categories where clinical signs, anatomical sites, and disease processes overlapped. Nevertheless, in the absence of a nationwide feline morbidity surveillance program in China, insurance claims provide a valuable and practical source of population-level descriptive evidence.

Another limitation is the definition of disease episodes. Because this study covered many diagnostic categories, a single rule was needed to distinguish new disease events from follow-up claims. Repeated claims within two weeks were, therefore, treated as recording the same disease episode in order to reduce the over-counting of repeat visits. This pragmatic approach is, we think, a justified simplification for a study with the aim of detailing broad morbidity patterns, but it is not suitable for all diseases. Acute conditions can often recur within a two-week period. In such a case, our approach would undercount the number of new disease episodes. Additionally, time to recurrence does not just depend on the disease but on the affected individual animals. Disease-specific studies using tailored look-back windows combined with clinical validation are better suited to investigating individual disorders in greater detail.

## 5. Conclusions

This study provides the first broad insurance-based description of morbidity patterns among insured cats in China using data from a major pet insurance provider. Digestive and urinary disorders accounted for the greatest morbidity burden, followed by infectious, respiratory, and dermatological disorders, with gastroenteritis and lower urinary tract disease being the most common diagnostic subcategories. Morbidity patterns varied by age, sex, and breed, with the highest overall incidence rate in cats less than one year of age and a higher burden of urinary disorders in male cats. Despite the limitations inherent to insurance claim data, these findings provide useful baseline evidence for veterinary practitioners, insurers, and researchers, and highlight the need for future disease-specific studies supported by more detailed clinical information.

## Figures and Tables

**Table 1:** Distribution of breed, sex, and neutering status, with incidence rate (IR) and 95% confidence interval (95%CI).

	Number of Cats ^1^		Number of Events ^2^	Time-at-Risk	Incidence Rate per 10,000 Cat-Years		
					IR	95%CI	
Breed							
Devon Rex	7925	4.8%	3100	9396	3299	3183	3415
Maine Coon	9421	5.7%	3494	11,147	3135	3031	3238
British Shorthair	146,544	89.4%	36,273	173,741	2088	2066	2109
Sex							
Male	102,045	62.3%	29,193	120,954	2414	2386	2441
Female	61,845	37.7%	13,674	73,330	1865	1833	1896
Neutering status							
Intact	95,933	58.5%	25,914	114,053	2272	2244	2300
Neutered	67,957	41.5%	16,953	80,230	2113	2081	2145

1, The total number of cats is 163,890. 2, The total number of disease events is 42,867.

**Table 2:** Number of disease events, incidence rate (IR) per 10,000 cat-years (total 194,284 cat-years from 24 September 2023 to 25 February 2025), 95% confidence interval (95%CI), and proportional morbidity for diagnostic categories.

Diagnostic Categories	Number of Events ^1^	Incidence Rate per 10,000 Cat-Years			Number of Cats	Proportional Morbidity ^2^
		IR	95%CI			%
Digestive	12,507	644	632	655	10,872	34.1%
Urinary	8906	458	449	468	7576	23.8%
Infectious disease	4320	222	216	229	3966	12.4%
Respiratory	4033	208	201	214	3508	11.0%
Dermatological	3912	201	195	208	3450	10.8%
Trauma	2054	106	101	110	1951	6.1%
Non-Specific Clinical Signs	1651	85	81	89	1584	5.0%
Ocular	1556	80	76	84	1402	4.4%
Otic	810	42	39	45	742	2.3%
Oral	788	41	38	43	752	2.4%
Reproductive	487	25	23	27	467	1.5%
Immunological	465	24	22	26	445	1.4%
Cardiovascular and Hematologic	465	24	22	26	435	1.4%
Musculoskeletal	368	19	17	21	354	1.1%
Neoplasia	247	13	11	14	241	0.8%
Neurological	200	10	9	12	183	0.6%
Metabolic and Endocrine	98	5	4	6	93	0.3%

1, The total number of events is 42,867. 2, The proportion of cats with specific diagnostic category relative to the total number of unique cats (31,870 cats). Since the same cat may suffer from multiple diseases, the sum of the number of cats is more than 31,870, and the sum of proportional morbidity is more than 100%.

**Table 3:** Number of disease events, incidence rate (IR) per 10,000 cat-years (total 194,284 cat-years from September 2023 to February 2025), 95% confidence interval (95%CI), and proportional morbidity for diagnostic subcategories.

Diagnostic Category	Diagnostic Subcategory	Number of Events	Incidence Rate per 10,000 Cat-Years			Number of Cats	Proportional Morbidity ^1^
			IR	95%CI			%
Digestive	Gastroenteritis	7119	366	358	375	6620	60.9%
	Pathogen	1722	89	84	93	1602	14.7%
	Digestive Dysfunction	708	36	34	39	696	6.4%
	Vomiting	601	31	28	33	591	5.4%
	Pancreatitis	390	20	18	22	373	3.4%
	Obstruction	381	20	18	22	369	3.4%
	Constipation	345	18	16	20	331	3.0%
	Diarrhea	221	11	10	13	218	2.0%
	Biliary	180	9	8	11	171	1.6%
	Hepatic Dysfunction	163	8	7	10	154	1.4%
	Hepatitis	133	7	6	8	129	1.2%
	Hepatic Lipidosis	63	3	2	4	61	0.6%
	Perianal	61	3	2	4	58	0.5%
	Hematochezia	52	3	2	3	51	0.5%
	Other GI	368	19	17	21	353	3.2%
Urinary	Lower Urinary	8420	433	424	443	7194	95.0%
	Upper Urinary	486	25	23	27	455	6.0%
Infectious Disease	FCV (Feline Calicivirus Infection)	1248	64	61	68	1202	30.3%
	FIP (Feline Infectious Peritonitis)	1016	52	49	56	938	23.7%
	FHV-1 (Feline Herpesvirus Type 1, Feline Viral Rhinotracheitis)	786	40	38	43	753	19.0%
	Mycoplasmosis	724	37	35	40	693	17.5%
	FPV (Feline Panleukopenia Virus)	500	26	23	28	493	12.4%
	Unspecified Viral Disease	35	2	1	2	34	0.9%
	FeLV (Feline Leukemia)	11	1	0	1	9	0.2%
Respiratory	Lower Respiratory	1484	76	72	80	1398	39.9%
	Upper Respiratory	1306	67	64	71	1224	34.9%
	Pneumonia	664	34	32	37	618	17.6%
	Tracheitis	477	25	22	27	453	12.9%
	Other Respiratory	102	5	4	6	100	2.9%
Dermatological	External Infections	2147	111	106	115	1967	57.0%
	Endogenous opportunistic Infections	563	29	27	31	546	15.8%
	Allergic	537	28	25	30	490	14.2%
	Unspecified Dermatitis	325	17	15	19	307	8.9%
	Pododermatitis	132	7	6	8	129	3.7%
	Ectoparasites	65	3	3	4	65	1.9%
	Other Dermatological Conditions	143	7	6	9	142	4.1%
Trauma	Fall/Vehicle Trauma	692	36	33	38	676	34.6%
	Toxic Ingestion	707	36	34	39	675	34.6%
	Bite/Abrasion/Soft Tissue Injury	540	28	25	30	524	26.9%
	Other Trauma	115	6	5	7	114	5.8%
Non-Specific Clinical Signs	Inflammatory Signs	520	27	24	29	505	31.9%
	Undiagnosed	333	17	15	19	332	21.0%
	Anorexia	245	13	11	14	242	15.3%
	Stress	128	7	5	8	125	7.9%
	Lameness	99	5	4	6	98	6.2%
	Anemia/Jaundice	83	4	3	5	80	5.1%
	Cavitary Effusions and Masses	81	4	3	5	78	4.9%
	Other signs	162	8	7	10	161	10.2%
Ocular	Keratoconjunctivitis	953	49	46	52	863	61.6%
	Periocular Disorders	252	13	11	15	242	17.3%
	Uveal	151	8	7	9	145	10.3%
	Chlamydial Infection	140	7	6	8	139	9.9%
	Other Ocular Disorders	60	3	2	4	56	4.0%
Otic	Otitis Externa	353	18	16	20	322	43.4%
	Otitis Media/Interna	193	10	9	11	186	25.1%
	Ear Mites	156	8	7	9	155	20.9%
	Aural Hematoma	59	3	2	4	56	7.5%
	Other Otic Disorders	49	3	2	3	49	6.6%
Oral	Stomatitis	278	14	13	16	266	35.4%
	Periodontal Disorders	267	14	12	15	261	34.7%
	Oral Trauma	144	7	6	9	136	18.1%
	Endodontic Disorders	52	3	2	3	52	6.9%
	Salivary Disorders	25	1	1	2	24	3.2%
	Other Oral Disorders	22	1	1	2	22	2.9%
Reproductive	Pyometra/Hydrometra/Endometritis ^2^	331	71	63	78	317	67.9%
	Ovarian/Vaginal/Mammary Disorders	66	3	3	4	64	13.7%
	Reproductive Emergencies and Pregnancy Disorders ^2^	47	10	7	13	47	10.1%
	Male Reproductive Disorders ^2^	30	4	3	6	30	6.4%
	Other Reproduction	13	1	0	1	13	2.8%
Immunological	Allergy	374	19	17	21	359	80.7%
	Lymph Node Disorders	48	2	2	3	47	10.6%
	Autoimmune Disease	31	2	1	2	28	6.3%
	Other Immunology	12	1	0	1	12	2.7%
Cardiovascular and Hematologic	HCM (Hypertrophic Cardiomyopathy)	169	9	7	10	165	37.9%
	Structural Heart Disease	120	6	5	7	115	26.4%
	Thrombosis/Coagulopathy	42	2	2	3	41	9.4%
	Pericardial Effusions/Chylothorax	34	2	1	2	31	7.1%
	Other Cardiovascular and Hematologic	100	5	4	6	95	21.8%
Musculoskeletal	Fracture	135	7	6	8	134	37.9%
	Arthritis	76	4	3	5	73	20.6%
	Hip Joint Disorders	49	3	2	3	48	13.6%
	Articular and Ligamentous Disorders	43	2	2	3	39	11.0%
	Muscle Disorders	24	1	1	2	24	6.8%
	Spinal Disorders	21	1	1	2	21	5.9%
	Other Musculoskeletal	20	1	1	1	18	5.1%
Neoplasia	Neoplasia	247	13	11	14	241	100%
Neurological	Epilepsy	62	3	2	4	53	29.0%
	Other Neurological	138	7	6	8	131	71.6%
Metabolic and Endocrine	Metabolic and Endocrine	98	5	4	6	93	100%

1, The proportion of cats with specific diagnostic subcategory relative to the diagnostic category number of unique cats. 2, For pyometra and reproductive emergencies, the at-risk population included only intact female cats (46,758 cat-years). For male reproductive disorders, the at-risk population included only intact male cats (67,295 cat-years).

**Table 4:** Comparison across studies of modified incidence rate (mIR) per 10,000 cat-years and proportional morbidity (PM) in cats for digestive and urinary disorders based on insurance claim data.

Reference	Country	Digestive		Urinary		All Disorders
		mIR ^1^	PM ^2^	mIR	PM	mIR
Current study	China	560	34.1%	390	23.8%	1640
Egenvall et al. (2010)	Sweden	144	16% ^3^	150	18% ^3^	875
Hadar et al. (2023)	Sweden	215	15%	196	13.8% ^3^	1430
Inoue et al. (2016)	Japan	1172	25.3%	1091	23.6%	4632
Isomura et al. (2017) ^4^	Japan	1120	22.7%	1120	22.7%	4940

1, Modified incidence rate was calculated as the number of cats with at least one disease event divided by time-at-risk in order to make the figures comparable across the references. 2, The proportion of cats with a specific diagnostic category relative to the total number of unique cats. 3, Different diagnostic categories are calculated by summation. 4, This reference reports annual prevalence, which is contextually comparable to the incidence rate per 10,000 cat-years. However, they should not be directly equated.

## Data Availability

The data presented in this study are available on request from the corresponding author. The data are not publicly available due to privacy or ethical restrictions.

## Supplementary Materials

The following supporting information can be downloaded at: https://www.mdpi.com/article/10.3390/vetsci13080817/s1, Table S1. Breed-specific incidence rate (IR) per 10,000 cat-years (total 194,284 cat-years from September 2023 to February 2025) and 95% confidence interval (95%CI) for diagnostic categories. Table S2. Classification dictionary of the hierarchical diagnostic classification system.
