# Supplementary material for: A Descriptive Analysis of Morbidity Patterns Among Insured Pet Cats in China Based on Insurance Claims
Source: Vet Sci. 2026 Aug 17;13(8):817. doi: 10.3390/vetsci13080817 (PMC13517770; doi:10.3390/vetsci13080817)
Supplement: Supplementary file 1 [file vetsci-13-00817-s001.zip › vetsci-4454436-supplementary.pdf]

Table S1. Breed-specific incidence rate (IR) per 10,000 cat-years (total 194,284 cat-years from September 2023 to February 2025) and 95% confidence interval (95%CI) for diagnostic categories.

Diagnostic Categories	Breed	Number of cases	Incidence rate per 10,000 cat-years		
			IR	95%CI	
Cardiovascular and Hematologic	Devon Rex	22	23	14	33
	Maine Coon	38	34	23	45
	British Shorthair	405	23	21	26
Digestive	Devon Rex	1010	1075	1009	1141
	Maine Coon	899	807	754	859
	British Shorthair	10,598	610	598	622
Immunological	Devon Rex	65	69	52	86
	Maine Coon	28	25	16	34
	British Shorthair	372	21	19	24
Infectious disease	Devon Rex	448	477	433	521
	Maine Coon	540	484	444	525
	British Shorthair	3332	192	185	198
Metabolic and Endocrine	Devon Rex	5	5	1	10
	Maine Coon	2	2	0	4
	British Shorthair	91	5	4	6
Musculoskeletal	Devon Rex	21	22	13	32
	Maine Coon	65	58	44	72
	British Shorthair	282	16	14	18
Neoplasia	Devon Rex	10	11	4	17
	Maine Coon	15	13	7	20
	British Shorthair	222	13	11	14
Neurological	Devon Rex	26	28	17	38
	Maine Coon	18	16	9	24
	British Shorthair	156	9	8	10

Non-Specific Clinical Signs	Devon Rex	125	133	110	156
	Maine Coon	135	121	101	142
	British Shorthair	1391	80	76	84
Ocular	Devon Rex	154	164	138	190
	Maine Coon	147	132	111	153
	British Shorthair	1255	72	68	76
Oral	Devon Rex	15	16	8	24
	Maine Coon	78	70	54	86
	British Shorthair	695	40	37	43
Otic	Devon Rex	45	48	34	62
	Maine Coon	149	134	112	155
	British Shorthair	616	35	33	38
Reproductive	Devon Rex	74	79	61	97
	Maine Coon	58	52	39	65
	British Shorthair	355	20	18	23
Respiratory	Devon Rex	312	332	295	369
	Maine Coon	519	466	426	506
	British Shorthair	3202	184	178	191
Skin	Devon Rex	421	448	405	491
	Maine Coon	297	266	236	297
	British Shorthair	3194	184	177	190
Trauma	Devon Rex	169	180	153	207
	Maine Coon	251	225	197	253
	British Shorthair	1634	94	89	99
Urinary	Devon Rex	178	189	162	217
	Maine Coon	255	229	201	257
	British	8473	488	477	498

Shorthair

Table S2. Classification dictionary of the hierarchical diagnostic classification system.

Diagnostic Category (Level 1)[1]	Diagnostic Subcategory (Level 2)	Key words for Diagnostic Category[2] or Diagnostic Subcategory[3]
Neoplasia		Disease contains '肿瘤|肥大细胞瘤|瘤|淋巴瘤|癌'<br>or Description contains '肿瘤|肥大细胞瘤' and Disease excludes '齿龈瘤|嗜酸性肉芽'
	Neoplasia	
Trauma		(Disease contains '指甲断裂|车祸|创伤|异物|外伤|创伤性皮肤病|烫伤|擦伤|误食|意外吃到鸡骨头|咬伤|创伤|戳伤|吸入性肺炎|挫伤|中毒|高楼|坠楼|食物中毒|眼球摘除|蹭伤|撕裂|下巴破溃||气胸|脚指|尾巴坏死|损伤|撞|细菌性化脓性感染|扭伤|摔伤|意外|夹伤|烧伤|舔到消毒液流涎|感染创|指甲脱落|膈破裂|^血肿$\|被针刺入\|昆虫叮咬\|蚊虫叮咬\|考虑被毒虫叮咬\|指甲发炎\|药物不良反应\|指甲根部折断\|指尖化脓\|指尖破溃\|断甲\|趾甲疾病\|骨渣便\|口吐白沫\|异常磨损\|吞食榴莲核\|吞食针线\|吞针' or Description contains '误食\|意外吞食\|异物\|车撞\|车祸\|坠楼\|坠落\|摔伤\|打架\|被咬\|楼掉下\|撞断\|跳楼\|坠楼\|中毒\|殴打\|扭到\|扭伤\|受伤\|崴\|腿骨错位\|摔.*跤\|摔到\|摔倒\|摔下\|高处\|拉伤\|撞到\|抓伤\|中毒\|受伤\|意外，被车碾压\|从楼上掉下\|掉下\|从五楼跳下\|^意外$|^外伤$\|摔落\|跳窗\|楼.*掉\|从.*楼\|意外掉落\|被其他狗咬伤，\|猫咪打翻了洗洁精\|嘴巴可能被自己咬到出血\|外伤，花费 2340 元\|鱼线鱼钩\|摔到受惊住院') and Disease excludes '子宫\|隐睾\|阴道炎\|疑似过敏性中毒癫痫\|腰椎脊柱炎\|炎性肠病\|牙周病\|牙髓炎\|牙吸收\|待查\|脓藓\|脓癣\|猫藓\|猫癣\|膀胱炎\|毛球症\|杯状\|猫瘟\|马拉色菌\|发育\|^胃肠炎$|胰|异位性皮炎|角膜溃疡|胃炎|过敏|急性肾损伤|肝|胃|肠|胃溃疡|眼睑内翻|肾功能损伤|肺炎|膀胱|寄生虫|肾|角膜损伤／刺激|脑损伤|外耳炎|皮肤病|尿|便秘|气管|呼吸道|肿大|中耳炎|息肉|口|食道|消化'<br>or Disease contains '细菌感染|细菌过度增殖|细菌性感染' and Description contains '破|伤口' and Description excludes '耳|眼|藓|癣|皮肤' and Disease excludes '急性肾损伤|肝|角膜溃疡|胃|肠|胃溃疡|眼睑内翻|肾功能损伤|肺炎|膀胱|寄生虫|肾|角膜损伤／刺激|脑损伤|外耳炎|皮肤病|尿|便秘|胰|气管|呼吸道|肿大|中耳炎|息肉|口|食道|消化'<br>or Disease includes '外科感染|绝育伤口出血|伤口感染 'and Description excludes '耳|眼|藓|癣|皮肤|尿|脾'<br>or Disease contains '胃内异物|胃部异物|^肠内异物$\|肠道异物\|误食\|^异物$|消化道异物|异物性肠梗阻|胃肠道异物|胃肠道梗阻|异物性肠炎|肠道异物性梗阻|胃内肠道异物|异物性胃肠炎|中毒.*肝|口吐白沫|鼻气管异物|消化异常''
	Fall/Vehicle Trauma	(Disease contains '高楼|坠楼|创伤|车|撞|夹|摔|高坠' or Description

<table>
<tr><td></td><td></td><td>contains '楼|车|撞|夹|摔|掉下|高处|跳') and Description excludes' 咬伤|打架|冲突'</td></tr>
<tr><td></td><td>Toxic Ingestion</td><td>Disease contains '异物|误食|肠梗阻|呕吐|口吐白沫|消化|吞食|中毒|蛇|巧克力|消毒液'
or Description contains '巧克力|中毒'</td></tr>
<tr><td></td><td>Bite/Abrasion/Soft Tissue Injury</td><td>Disease contains '咬伤|外伤|损伤|感染|撕裂|蹭伤|擦伤|挫伤|肌|软组织|舌|组织挫伤|气胸'
or Description contains '咬伤|抓伤|创伤|意外|撕|蹭伤|擦伤'</td></tr>
<tr><td></td><td>Other Trauma</td><td></td></tr>
<tr><td>Infectious Disease</td><td></td><td>Disease contains '杯状|惰性溃疡|猫传染性腹膜炎|猫瘟|疱疹|猫鼻支|猫传染性鼻气管炎|猫鼻支气管炎|泛白细胞|白血病病毒|支原体|传腹|传染性腹膜炎|细小|犬腺病毒|犬瘟|传染性肝炎|尿毒症|重度肾衰|乳酸中毒 心脏肿瘤 贫血、乳酸中毒低血氧症|传染病|白血病' and Disease excludes '^猫龈口炎$|牙龈炎|淋巴瘤|杯状病毒性口炎|^肺炎$|鼻炎|呼吸道|鼻窦炎|角膜|结膜|膀胱结石|肠炎|肺|短头|咳血|呼吸|倒吸|心|胸腔|病毒性肠胃炎|鼻泪管'
or Description contains '检查后是猫瘟|^杯状$|猫鼻支|感染猫疱疹和杯状病毒' and Disease excludes'^猫龈口炎$|牙龈炎|淋巴瘤|杯状病毒性口炎|^肺炎$|鼻炎|鼻窦炎|角膜|结膜|膀胱结石|肠炎|肺部|肺叶|肺泡|肺塌陷|短头|咳血|呼吸|倒吸|心|胸腔|病毒性肠胃炎|鼻泪管'
or Description contains '因眼睛红肿到百诺'</td></tr>
<tr><td></td><td>FCV
(Feline Calicivirus Infection)</td><td>Disease contains '杯状'
or Disease contains '杯状|传染病|惰性溃疡' and Description excludes '猫鼻支|鼻支|传染性鼻|杯状'</td></tr>
<tr><td></td><td>FIP
(Feline Infectious Peritonitis)</td><td>Disease contains '传染性腹膜炎|传腹|冠状'
or Disease contains '^传染病$' and Description contains '冠状'</td></tr>
<tr><td></td><td>FHV-1
(Feline Herpesvirus Type 1, Feline Viral Rhinotracheitis)</td><td>Disease contains '疱疹病毒|猫鼻支|传鼻|传染性鼻气管'
or Description contains '猫鼻支|鼻支|疱疹|fhv'</td></tr>
<tr><td></td><td>Mycoplasmosis</td><td>Disease contains '支原体|传贫|传染性贫血'
or Disease contains '传染病|病毒性传染病' and Description contains '支原体|流泪|结膜|角膜|眼睛|传贫|传染性贫血'</td></tr>
<tr><td></td><td>FPV
(Feline Panleukopenia Virus)</td><td>(Disease contains '猫瘟|猫泛白细胞减少症|细小' or Description contains'^传染病$') and Description contains '猫瘟|细小'</td></tr>
<tr><td></td><td>FeLV
(Feline Leukemia)</td><td>Disease contains '白血病'</td></tr>
<tr><td></td><td>Unspecified Viral Disease</td><td></td></tr>
<tr><td>Digestive</td><td></td><td>Disease contains '消化系统|消化功能|消化吸收功能|消化性溃疡|肠|</td></tr>
</table>

	胃|消化道|肠道|结肠|滴虫|贾第|肠道寄生虫感染|冠状病毒|冠状病毒性肠炎|^冠状$\|腹膜炎\|星状病毒\|食物不耐受\|肝胆.*郁\|螺杆菌\|肠.*炎症\|戳伤\|消化不良\|肠梗阻\|毛球\|胰腺炎\|贲门\|幽门\|十二指肠溃疡\|球虫\|弯曲杆菌\|发烧，病毒感染\|返流\|痢疾\|消化功能紊乱\|猫粮不耐受\|隐孢子虫\|呼吸性碱中毒\|腹膜炎\|星状病毒\|产气荚膜菌感染\|腹壁疝\|腹股沟疝\|腹腔出血\|腹腔出血\|腹痛\|脐疝\|菌群\|酵母菌\|芽孢杆菌感染\|查帕哈马病毒感染\|IBD\|食物不良反应\|液体脓包' and Disease excludes '异物\|细小\|支气管炎,\|肿瘤\|瘤\|脾胃不和\|脾胃虚寒\|便秘\|淋巴瘤\|尖瓣\|主动脉\|肺\|牙龈' and Description excludes'意外吞食'<br>or Disease contains '冠状病毒感染\|猫冠状病毒感染\|猫肠道冠状病毒感染\|毛滴虫病\|螺杆菌感染\|梭状芽孢杆菌性肠炎\|贾第鞭毛虫病\|贾第虫病\|弯曲杆菌病\|球虫病\|滴虫病\|阿米巴病\|阿米巴虫病\|轮状病毒感染\|沙门氏菌病\|三毛滴虫感染\|胎儿三毛.*虫\|肠道寄生虫病\|蛔虫\|绦虫\|鞭毛虫\|^吸虫病$|^原虫病$\|寄生虫病\|寄生虫感染' and Description excludes '腹膜炎\|积液\|FIP\|传染性腹膜炎\|猫传染性腹膜炎\|传腹\|干性传腹\|湿性传腹腹膜炎\|积液\|FIP\|传染性腹膜炎\|猫传染性腹膜炎\|传腹\|干性传腹\|湿性传腹\|蜱虫\|.*尖瓣\|肺动脉瓣生理性返流\|牙'<br>or Disease contains'肝功能不全\|肝\|胆囊\|肝肾\|胆\|胰腺\|腰腺外分泌功能不全\|三体炎\|肝.*吸虫' and Disease excludes '肿瘤\|癌\|瘤\|糖尿病\|中毒\|肝门静脉\|肝性脑病\|总胆红素低\|.*尖瓣\|肺动脉瓣生理性返流\|牙龈'<br>or Disease contains'肛\|直肠\|^异物性肠胃炎$'<br>or Disease contains '呕吐|腹泻|吐血|便血|大便带血|血便|血性粘稠分泌物|拉稀|便秘|大便蓄积|排便困难|软便|功能性梗阻|积食|食道|膈疝|横膈膜疝|梭菌感染|急性阴性菌感染|博卡|消化功能紊乱|催吐|$返流^' and Description excludes '猫鼻支' and Disease excludes'主动脉|.*尖瓣|肺'<br>or Disease contains '食物不耐受' and Description contains '肠胃炎|胃肠炎'<br>or Disease contains '细菌感染|细菌过度增殖|细菌性感染|外科感染' and Description contains '呕吐|腹泻|拉稀|脾'
Gastroenteritis	Disease contains '胃肠疾病|胃肠炎|肠胃炎|急性肠炎|胃炎|结肠炎|肠炎|肠道|炎性肠病|胃肠感染|胃肠道疾病|胃肠综合征|胃肠型感冒|胃溃疡|溃疡|细菌性感染|消化道炎症|消化系统感染' and Disease excludes '积气|胀气|梗阻|毛球|异物|积液|病毒|菌|障碍|肠道划伤|肠道疾病|肝|肝吸虫|胃肠疾病|虫|不耐受|宿便|肉芽肿'
Pathogen	Disease contains '毛滴虫病|螺杆菌感染|螺旋|贾第鞭毛虫病|贾第虫病|弯曲杆菌病|球虫病|滴虫病|阿米巴病|阿米巴虫病|轮状病毒感染|沙门氏菌病|三毛滴虫感染|胎儿三毛滴虫|肠道寄生虫病|梭状芽孢杆菌性肠炎|滴虫|虫|幽门.*菌|隐孢子虫|寄生虫|细菌性|冠状病毒|传腹|传染性腹膜炎|腹膜炎|病毒|肝吸虫|芽孢杆菌感染|弯曲杆菌|急性阴性菌感染|感染|冠状|寄生虫'
Digestive	Disease contains '消化不良|紊乱|胀气|积液|积食|功能不良|动力|迟缓|

<table>
<tr><td></td><td>Dysfunction</td><td>功能障碍|障碍|肠道疾病|食物不耐受|功能|菌群失调|猫粮不耐受|食物不良反应|肠胃不耐|不适|积气|积食|消化系统疾病|胃肠疾病' and Disease excludes '肝|胆|胰|梗阻'</td></tr>
<tr><td></td><td>Vomiting</td><td>Disease contains '呕吐'</td></tr>
<tr><td></td><td>Pancreatitis</td><td>Disease contains'胰腺炎'</td></tr>
<tr><td></td><td>Obstruction</td><td>Disease contains '毛球症|异物|误食|梗阻|巨结肠|阻塞|堵塞'</td></tr>
<tr><td></td><td>Constipation</td><td>Disease contains '便秘|排便困难|大便蓄积|肠道宿便'</td></tr>
<tr><td></td><td>Diarrhea</td><td>Description contains '腹泻|软便|拉稀'</td></tr>
<tr><td></td><td>Biliary</td><td>Disease contains '胆囊炎|胆汁淤积|胆管炎|胆' and Disease excludes '肝'</td></tr>
<tr><td></td><td>Hepatic Dysfunction</td><td>Disease contains '肝功能不全|肝.*功能|肝功|肝指标异常'</td></tr>
<tr><td></td><td>Hepatitis</td><td>Disease contains '肝炎|炎性肝|肝胆炎'</td></tr>
<tr><td></td><td>Hepatic Lipidosis</td><td>Disease contains '脂肪肝'</td></tr>
<tr><td></td><td>Perianal</td><td>Disease contains '肛|直肠'</td></tr>
<tr><td></td><td>Hematochezia</td><td>Disease contains '血' and Disease excludes '出血'</td></tr>
<tr><td></td><td>Other GI</td><td></td></tr>
<tr><td>Urinary</td><td></td><td>Disease contains '膀胱|肾|尿|结石|磷酸铵镁' and Disease excludes '淋巴瘤|糖尿病|肾上腺|心肾|牙结石|多饮多尿|血尿带查|尿血待查'
or Disease contains '细菌感染|外科感染' and Description contains '尿'</td></tr>
<tr><td></td><td>Lower Urinary</td><td>Disease contains '尿道梗阻|尿道炎|尿道结石|尿道感染|尿闭|尿道综合征|下泌尿道综合征|膀胱炎|膀胱结石|膀胱结晶|自发性膀胱炎|膀胱感染|血尿性膀胱炎|膀胱疾病|尿|膀胱|结晶|外科感染|细菌感染'
or Description contains '尿道梗阻|尿道炎|尿道结石|尿闭|导尿|尿道感染|尿道堵塞|膀胱疾病|膀胱炎|膀胱结石|膀胱结晶|血尿|膀胱肿大|膀胱感染' and Disease excludes '肾' and Description excludes '肠胃炎|胃肠炎|肠炎|胃炎|结肠炎|急性肠胃炎|急性肠炎|急性胃炎|慢性肠炎|急性出血性肠胃炎|细菌性肠胃炎|直肠炎|滴虫|滴虫感染|贾第鞭毛虫感染|螺杆菌|弯曲杆菌|球虫|冠状病毒|猫冠状病毒'</td></tr>
<tr><td></td><td>Upper Urinary</td><td>Disease contains '肾|肾脏|肾结石|肾衰|急性肾损伤|慢性肾衰|肾炎|肾功能衰竭|尿毒症|输尿管'
or Description contains '肾结石|肾衰|肾损伤|肾功能|尿毒症|肾炎|输尿管' and Disease excludes '尿道|膀胱' and Description excludes '肠胃炎|胃肠炎|肠炎|胃炎|结肠炎|急性肠胃炎|急性肠炎|急性胃炎|慢性肠炎|急性出血性肠胃炎|细菌性肠胃炎|直肠炎|滴虫|滴虫感染|贾第鞭毛虫感染|螺杆菌|弯曲杆菌|球虫|冠状病毒|猫冠状病毒'</td></tr>
<tr><td>Respiratory</td><td></td><td>Disease contains '肺炎|支气管肺炎|肺水肿|肺部感染|猫哮喘|支气管败血波氏杆菌
or Disease contains '呼吸系统疾病|咳嗽|呼吸急促|打喷嚏|流鼻涕|呼吸道|感冒' and Description contains '肺炎|支气管肺炎|肺水肿|肺部感染' and Disease excludes '牙|心|心肌|结膜|角膜|耳|鼻泪管|口炎|皮肤|皮炎|猫癣|尖瓣|动脉|耳螨|鼓室|白内障|表皮囊肿|腹腔|关节|疥螨|脑|葡萄膜|口腔|免疫|食'</td></tr>
</table>

		or Disease contains '慢性支气管炎|过敏性支气管炎|支气管炎|呼吸系统疾病|鼻炎|鼻涕|窦炎|鼻窦|额窦|鼻腔|猫鼻支|上呼吸道感染|呼吸道感染|猫杯状病毒感染|猫疱疹病毒感染|支原体感染|波氏杆菌感染|咳嗽|打喷嚏|鼻塞|猫传染性鼻气管炎|鼻息肉|鼻咽狭窄|鼻黏膜|鼻粘膜|鼻渊|鼻子敏感|鼻子倒吸气|鼻孔狭窄|鼻部急性出血|$芽孢杆菌^'<br>or Disease contains '呼吸系统疾病\|咳嗽\|呼吸急促\|打喷嚏\|流鼻涕\|呼吸道\|感冒\|感染'and Description contains '鼻炎\|猫鼻支\|上呼吸道\|打喷嚏\|流鼻涕\|鼻塞\|咳嗽\|呼吸道感染\|支原体\|杯状病毒\|疱疹病毒\|呼吸道疾病' and Disease excludes '牙\|心\|心肌\|结膜\|角膜\|耳\|鼻泪管\|口炎\|皮肤\|皮炎\|猫癣\|尖瓣\|动脉\|耳螨\|鼓室\|白内障\|表皮囊肿\|腹腔\|关节\|疥螨\|脑\|葡萄膜\|口腔\|免疫\|食'<br>or Disease contains '呼吸系统疾病\|呼吸道\|呼吸\|支气管炎\|气管\|肺\|波氏\|波士\|犬副流感\|鼻炎\|哮喘\|鼻窦\|合胞体\|咽喉炎\|脓胸\|咳血\|咽喉部\|咽炎\|喉部疾病\|喉炎\|咽喉部\|猫查帕哈马病毒\|博代氏杆菌\|胸膜炎\|扁桃体炎\|嗜麦芽窄食单胞菌感染\|查帕哈马病毒\|$芽孢杆菌^|短头品种气道|倒吸|痰栓' and Disease excludes '猫鼻支|疱疹|支原体|肾|淋巴瘤|肥厚型心肌病|肿瘤|癌|肺心病|心肺|异物|肺动脉|杯状|腹式呼吸|脉|血管|应激|呼吸性碱中毒|猫传染性鼻气管炎|猫鼻支气管炎|牙|心|心肌|结膜|角膜|耳|鼻泪管|口炎|皮肤|皮炎|猫癣|尖瓣|动脉|耳螨|鼓室|白内障|表皮囊肿|腹腔|关节|疥螨|眼|脑|葡萄膜|口腔|免疫|食'<br>or Disease contains '咳嗽|鼻炎|$芽孢杆菌^|' and Description excludes '呼吸道|肺|气管' and Disease excludes '牙|心|心肌|结膜|角膜|耳|鼻泪管|口炎|皮肤|皮炎|猫癣|尖瓣|动脉|耳螨|鼓室|白内障|表皮囊肿|腹腔|关节|疥螨|眼|脑|葡萄膜|口腔|免疫|食'
	Lower Respiratory	Disease contains '呼吸道感染|呼吸道疾病|下呼吸道|呼吸系统疾病|气管|胸|肺水肿|肺|倒吸|呼吸道症状|呼吸道炎症|气管|哮喘|波氏杆菌|病毒|菌|衣原体|感染'
	Upper Respiratory	Disease contains '上呼吸道感染综合征|咳嗽|鼻|鼻窦炎|呼吸困难|咽喉炎|上呼吸道|额窦|喉|咽|短头|感冒|喷嚏'
	Pneumonia	Disease contains '肺炎'
	Tracheitis	Disease contains '气管炎'
	Other Respiratory	
Dermatologica<br>l		Disease contains '皮肤|猫藓|猫癣|癣菌|藓菌|癣菌感染|真菌感染|脓癣|湿疹|脓皮病|皮下脓肿|颌下囊肿|颈部囊肿|脸下囊肿|^囊肿$\|囊肿切开\|峡部囊肿\|指间\|混合感染\|过敏性皮炎\|肛门周边炎症\|肉芽肿\|皮炎\|甲沟炎\|真菌\|毛囊\|皮脂腺\|尾腺增生\|尾部\|尾根发炎\|尾巴深度化脓性感染\|球菌\|孢子菌\|猫特应性综合征\|蜂窝织炎\|丘疹\|脓藓\|荨麻疹\|痤疮\|疖\|细菌性足炎\|脚垫细菌感染\|脓肿\|表皮\|皮下囊肿\|脱毛\|脓皮\|脓藓\|跳蚤\|脱水\|皮下脓肿\|皮脂\|皮表肿物\|皮样囊肿\|脆皮病\|类皮窦\|皮赘\|皮下化脓感染\|蜂窝\|鼻部色素减退\|马拉色菌\|黑下巴\|寄生虫叮咬\|化脓.*感染\|细菌性足炎\|足底破溃\|瘙痒\|念珠菌病\|浅表化脓杆菌\|乳头发炎\|体表增生破溃感染\|下颌感染\|嘴角息肉\|角化\|体表'<br>or Description contains '^皮肤病$' and Disease excludes '肿瘤|淋巴瘤|

		皮肤外伤|创伤性皮肤病|(猫疱疹病毒 1 型)|肥大细胞瘤|瘤|烫伤|擦伤|内分泌性皮肤病|肠道寄生虫感染、皮肤病|螨虫中耳炎|疱疹支原体|脑室|肠道|胃炎|耳道|耳炎' and Description excludes '肿瘤|奶黄|耳'<br>or Disease contains'螨|蜱' and Disease excludes '耳'<br>or Disease contains '细菌感染|细菌过度增殖|细菌性感染|细菌性脓皮疹|细菌性脓皮症|外科感染' and Description contains '猫藓|猫癣|皮肤|足|脚|脱毛'<br>or Disease contains '趾间|指.*间囊肿|犬指(趾)间囊肿|浆细胞性趾间皮炎|浆细胞性足炎|甲床|脚垫|脚指'<br>or Description contains '趾间炎|蜱'
	External Infections	Disease contains '猫藓|猫癣|细菌|真菌|癣菌|藓菌|真茵|癣茵|藓茵|孢子菌|小孢子菌|混合'<br>or Disease contains '皮肤病|皮炎|自损性皮炎|皮肤瘙痒症' and Description contains '猫藓|猫癣|细菌|真菌|皮肤癣菌|孢子菌|小孢子菌' and Disease excludes '马拉色|嗜酸性|特应性|特异性'
	Endogenous Opportunistic Infections	Disease contains '脓皮|脓肿|毛囊炎|化脓性|疖|球菌|葡萄|马拉色菌|马拉|念珠菌'<br>or Disease contains '皮肤病|皮炎|自损性皮炎|皮肤瘙痒症' and Description contains '马拉色菌|疖'
	Allergic	Disease contains '过敏|特异性皮炎|特发性皮炎|天疱疮|异位性皮炎|特应性|特发性面部皮炎|浆细胞性皮炎|湿疹|嗜酸性肉芽肿|免疫|蚊蝇|荨麻疹|褶皱性|叮咬|嗜酸性'
	Unspecified Dermatitis	Disease contains '皮肤病|皮炎|瘙痒|皮肤炎|丘疹|痤疮|脂'
	Pododermatitis	Disease contains '趾|指|足|脚|甲|爪|肉垫'<br>or Disease contains '皮肤病|皮炎|自损性皮炎|皮肤瘙痒症' and Description contains '趾|指|足|脚|甲沟炎|爪|肉垫'
	Ectoparasites	Disease contains '跳蚤|寄生虫|螨'<br>or Disease contains '皮肤病|皮炎|自损性皮炎|皮肤瘙痒症' and Description contains '跳蚤|寄生虫|螨'
	Other Dermatological Conditions	
Metabolic and Endocrine	Metabolic and Endocrine	Disease contains '内分泌|肾上腺|甲状|甲减|甲亢|糖尿病|假孕|钙|高.*症|代谢|肝性脑病|^营养不良$|钾|钠|库欣|高血糖|低血糖|磷血|镁血|肥胖症|微量元素缺乏|艾迪森|甘油三酯升高|电解质|营养缺乏' and Disease excludes '肺|胆红素|高炎症'
Non-Specific Clinical Signs		Disease contains '风湿热郁|^呕吐$\|^腹泻$|病因待查|脾胃不和|脾胃虚寒|脾气虚|气滞证|喷嚏|发热|无名|高热|心肺骤停|心肺待查|抽搐待查|不明|待定|厌食|食物不耐受|应激|食欲|跛|休克|黄疸|贫血|气胸|心脏骤停|感冒|总胆红素低|伤口感染|根周感染|血清肿|特发性营养不

<table>
<tr><td></td><td></td><td>良|呼吸困难|腹式呼吸|食欲.*待查|嗜睡.*待查|转诊.*待查|疼痛.*|胸腔|腹水|脱水|厌氧|疑似传腹|待查|氮质血症|皮下积液|皮下增生|皮下肿物|皮下肿块|皮下血肿|喉头|趾根断裂|.*肢无力|后肢检查|.*肢肿胀|后肢感染|左后肢炎性水肿|前肢水肿|高体温|肿物|肿块|腹部肿块|腹围增大|组织囊肿|特发性弥散性水肿|局部水肿|外周水肿|炎性水肿|气血两虚|阴虚血虚|低血容量|脂膜炎|行为问题|行为异常|受凉|外周水肿|低烧|总蛋白偏高|未见.*异常|^病毒感染$|感染|.*肢肿胀|炎性增生|炎性肿胀|^炎性$|猝死|发烧|高烧|过度理毛|抑郁|异嗜|复查|低体温|抵抗力低|总胆红素低|急性感染|慢性感染|^感染$|消瘦|运动不耐受|情绪|生理性抖动|后腿无力|精神|占位性|突发性无力|^增生$|晕|喘气症|抗体|空气性风寒|双腿后支无力|^流感$|^猫流感$|颅内压升高|右侧前段肿胀|胸水|面部肿胀|慢性消耗病|多饮多尿|高炎症|中暑|日射病|热射病|多器官衰竭|败血症|肝性脑病' and Disease excludes '肠|胃炎|膀胱炎|胰腺|肺水肿|免疫介导性.*血'
or Disease contains'蛋白血症|鼻出血|抽搐|流涎|流口水|毛细血管破裂|狂犬免疫|通融赔付'
or Disease contains'炎症' and Disease excludes '肠|耳|子宫|下泌尿道|耳道|脾|眼睛|肛门'
or Disease contains '细菌感染|细菌过度增殖|细菌性感染|病毒性感染|疑似病毒感染' and Description excludes '眼|耳'</td></tr>
<tr><td></td><td>Inflammatory Signs</td><td>Disease contains '炎症|高热|低烧|高烧|败血症|发热|高体温|发烧|感冒|感染炎性|感染|流感'
or Disease contains '病因待查' and Description contains '炎症|高热|低烧|高烧|败血症|发热|高体温|发烧|感冒|感染|炎性|感染'</td></tr>
<tr><td></td><td>Undiagnosed</td><td>Disease contains '病因待查|待定|复查|待查'</td></tr>
<tr><td></td><td>Anorexia</td><td>Disease contains '厌食|食欲|消瘦'
or Disease contains '病因待查' and Description contains '厌食|食欲|消瘦|不吃'</td></tr>
<tr><td></td><td>Stress</td><td>Disease contains '应激'
or Disease contains '病因待查' and Description contains '应激|惊吓'</td></tr>
<tr><td></td><td>Lameness</td><td>Disease contains '跛行|肿胀|水肿|肢|腿|站'
or Disease contains'病因待查|水肿|肿胀' and Description contains '跛行|肿胀|水肿|肢|腿|站'</td></tr>
<tr><td></td><td>Anemia/Jaundice</td><td>Disease contains '贫血|黄疸'
or Disease contains '病因待查' and Description contains '贫血|黄疸'</td></tr>
<tr><td></td><td>Cavitary Effusions and Masses</td><td>Disease contains '腹水|积液|积水|腹围|胸水|胸腔|腹腔|胸壁|腹壁|脾脏肿物'
or Disease contains '病因待查' and Description contains '腹水|积液|黄疸|积水|腹围|胸水|胸腔|腹腔'</td></tr>
<tr><td></td><td>Other signs</td><td></td></tr>
<tr><td>Ocular</td><td></td><td>Disease contains'结膜|角膜炎|鼻泪管|葡萄膜|葡膜|角膜|眼|睑板|衣原体|白内障|睫|睑|巩膜|前房|虹膜|视网膜|内眦|流泪' and Description excludes '前庭'</td></tr>
</table>

<table>
<tr><td></td><td></td><td>or Disease contains '细菌感染' and Description contains '眼'</td></tr>
<tr><td></td><td>Keratoconjunctivitis</td><td>(Disease contains '结膜炎|角膜溃疡|角膜炎|腐骨' or Disease contains '眼病|眼.*病' and Description contains '结膜炎|角膜溃疡|角膜炎|腐骨') and Description excludes '衣原体'</td></tr>
<tr><td></td><td>Periocular Disorders</td><td>Disease contains '睑|睫|鼻泪管'
or Disease contains '眼病|眼.*病' and Description contains '睑|睫|鼻泪管'</td></tr>
<tr><td></td><td>Uveal</td><td>Disease contains '葡|虹膜睫状体炎'
or Disease contains '眼病|眼.*病' and Description contains '葡|虹膜睫状体炎'</td></tr>
<tr><td></td><td>Chlamydial Infection</td><td>Disease contains '衣原体|结膜|角膜'
or Disease contains '眼病|眼.*病' and Description contains '衣原体'</td></tr>
<tr><td></td><td>Other Ocular Disorders</td><td></td></tr>
<tr><td>Reproductive</td><td></td><td>Disease contains '子宫|乳腺|乳房炎|前列腺|发情|阴道|外阴|卵巢|包皮|阴茎|生殖器|前列腺|睾丸|慢性良性腺体增生|阴门|阴囊|隐睾|难产|妊娠|死胎|不育|流产|孕检|产检|剖腹产|去势|产后|嵌顿包茎|炎性反应增生|分娩|胎盘滞留|死胎|性发育紊乱' and Disease excludes '膀胱
or Description contains '子宫蓄脓' and Disease excludes '胆囊'
or Disease contains '绝育' and Description contains '子宫'</td></tr>
<tr><td></td><td>Pyometra/Hydrometra/Endometritis</td><td>Disease contains '蓄脓|子宫.*脓|子宫.*液|内膜炎|子宫炎'
or Description contains '蓄脓|子宫.*脓|子宫.*液|内膜炎|子宫炎'</td></tr>
<tr><td></td><td>Ovarian/Vaginal/Mammary Disorders</td><td>Disease contains '卵巢|阴道|乳'</td></tr>
<tr><td></td><td>Reproductive Emergencies and Pregnancy Disorders</td><td>Disease contains '产|胎|妊娠|分娩|孕'</td></tr>
<tr><td></td><td>Male Reproductive Disorders</td><td>Disease contains '睾|公猫|包皮|阴囊'</td></tr>
<tr><td></td><td>Other Reproduction</td><td></td></tr>
<tr><td>Immunological</td><td></td><td>Disease contains '过敏|风湿|免疫|嗜酸性|淋巴腺|淋巴|天疱疮|盘状红斑狼疮|疫苗' and Disease excludes '风湿热郁|狂犬|第四脑室|贫血'</td></tr>
<tr><td></td><td>Allergy</td><td>Disease contains '过敏|免疫性疾病'</td></tr>
<tr><td></td><td>Lymph Node Disorders</td><td>Disease contains '淋巴'</td></tr>
<tr><td></td><td>Autoimmune Disease</td><td>Disease contains '白细胞|血小板|嗜酸性|关节炎|天疱疮'
or Description contains '白细胞|血小板|嗜酸性|关节炎|天疱疮'</td></tr>
<tr><td></td><td>Other Immunology</td><td></td></tr>
<tr><td>Otic</td><td></td><td>(Disease contains '耳|鼓|前庭|前厅|马拉色菌' or Description contains '耳|前庭') and Disease excludes '肿瘤|折耳猫骨骼疾病|咬伤|牙|口|脾|癫痫|心|骨|关节|腱|面神经|脑|神经'</td></tr>
</table>

		or Disease contains '细菌感染' and Description contains '耳'
	Otitis Externa	Disease contains '外耳炎|耳炎|外耳|过敏|异物|耳道发炎'<br>or Description contains '耳炎|外耳炎|细菌'
	Otitis Media/Interna	Disease contains '中耳炎|鼓室积脓|鼓室|积脓|内耳炎|前庭|眼球震颤|共济失调'<br>or Description contains '内耳炎|前庭|眼球震颤|共济失调|中耳炎'
	Ear Mites	Disease contains '螨|寄生虫'<br>or Description contains '螨'
	Aural Hematoma	Disease contains '耳血肿'
	Other Otic Disorders	
Musculoskeletal		Disease contains '折耳猫骨骼疾病|关节炎|腰椎脊柱炎|锥体病|腰|骨折|关节|椎|骨|肌肉|屈肌|咬肌|平滑肌|营养不良|韧带|肌腱|半月板|软骨|重症肌无力|脊柱|软组织|骨.*发育|肌|肘|腱|髋臼|脱臼|运动系统|^拉伤$|孟上结节撕脱' and Disease excludes '风湿性|免疫介导性|腰腺外分泌功能不全|左侧腰七骶一神经根受压|糖尿病|骨渣便|牙体|心|神经|脊髓|脊膜'
	Fracture	Disease contains '骨折|骨裂|后技骨新'
	Arthritis	Disease contains '关节炎'
	Hip Joint Disorders	Disease contains '髋|股'
	Articular and Ligamentous Disorders	Disease contains '韧带|软组织|脱位|关节|下颌骨错位|下颌骨闭锁|软骨|折耳|孟上结节'
	Muscle Disorders	Disease contains '肌|腱'
	Spinal Disorders	Disease contains '椎|锥'
	Other Musculoskeletal	
Cardiovascular and Hematologic		Disease contains '肺心病|肺动脉|心肺疾病|肝门静脉|心肌|心肾|心|动脉|无形体|埃|无浆体|尖瓣|乳糜胸|高血压|左室舒张功能下降|室间隔|瓣膜|室.*早搏|房间隔|肺静脉|尖瓣|反流' and Disease excludes '中心|心脏骤停'<br>or Disease contains '血小板|巴贝斯|焦虫|溶血|贫血|血液|凝血|血友病|血栓|紫癜|白血病|骨髓|嗜.*粒|淋巴细胞|血凝不良|出血性紫癜|寄生虫病|白球|白细胞' and Disease excludes '主动脉口|泛|白血病病毒|皮肤淋巴细胞样增生'<br>or Disease contains '脾'
	HCM (Hypertrophic Cardiomyopathy)	Disease contains '肥厚.*心肌|肥'<br>or Description contains '肥厚.*心肌'
	Structural Heart Disease	Disease contains '房|室|心脏|心肌|瓣膜|瓣|反流|返流'
	Thrombosis/Coagulopathy	Disease contains '血栓|溶血|凝血|血凝|血小板|紫癜'

<table>
<tr><td></td><td>Pericardial Effusions/Chylothorax</td><td>Disease contains '心包|乳糜胸'</td></tr>
<tr><td></td><td>Other Cardiovascular and Hematologic</td><td></td></tr>
<tr><td>Oral</td><td></td><td>Disease contains '牙|齿|口炎|口腔|嘴唇|舌|无痛性溃疡|^溃疡$|杯状病毒性口炎|口鼻瘘|口面瘘|咬合不正|唇|颜面瘘|软腭|龈|复杂冠折|牙齿撕脱|牙齿断裂|牙齿折断|牙齿损伤|齿折|犬齿断裂|齿体损伤' and Disease excludes '洁牙|洗牙'
or Disease contains '腮腺|唾液腺|舌下腺|下颌腺|颌下腺|颧腺|腺体过度分泌'</td></tr>
<tr><td></td><td>Stomatitis</td><td>Disease contains '口炎|溃疡|舌|黏膜|口腔发炎|唇|口腔炎|软腭'</td></tr>
<tr><td></td><td>Periodontal Disorders</td><td>Disease contains '牙周|牙龈|牙结石|齿龈炎|龈膜炎'</td></tr>
<tr><td></td><td>Oral Trauma</td><td>Disease contains '牙齿撕脱|牙齿断裂|牙齿折断|牙齿损伤|齿折|犬齿断裂|齿体损伤|外伤|异物'
or Description contains '外伤|意外|误食|吞|卡|扎'</td></tr>
<tr><td></td><td>Endodontic Disorders</td><td>Disease contains '牙髓|牙吸收|齿折|冠折|坏死|牙根|牙体骨折|拔牙|牙本质|龋齿|齿龈漏|齿龈增生'</td></tr>
<tr><td></td><td>Salivary Disorders</td><td>Disease contains '腺'</td></tr>
<tr><td></td><td>Other Oral Disorders</td><td></td></tr>
<tr><td>Neurological</td><td></td><td>Disease contains '脑|疑似过敏性中毒瘢痫|震颤|Chiari|霍纳|神经|脑|脊髓|癫痫|焦虑|左侧腰七骶一神经根受压|马尾|第四脑室肉芽肿|瘫痪|面瘫|猫瞳孔痉挛综合征|共济失调|中枢|脊膜'</td></tr>
<tr><td></td><td>Epilepsy</td><td>Disease contains '癫痫'
or Description contains '癫痫'</td></tr>
<tr><td></td><td>Other Neurological</td><td></td></tr>
</table>

1*, The diagnostic category order from top to bottom represents the classification priority sequence. Therefore, records that match keywords of multiple diagnostic categories are assigned to the higher-ranked diagnostic category.

2*, Includes Disease and Description columns. For conciseness, some R syntax has been retained: | represents "or"; the "and" operator has higher precedence than the "or" operator; the keyword '肿瘤' can be used to filter for terms such as '肿瘤', '肛周肿瘤', '肠道肿瘤', while the keyword '^肿瘤$' can only be used to filter for '肿瘤'; '从.*楼' can be used to filter for terms such as '从 8 楼', '从八楼', '从楼上'; inside parentheses has higher precedence than outside parentheses.

3*, Restrict to the corresponding diagnostic category.
